# Comprehensive and quantitative urinary metabolomic profiling for improved characterization of diabetic nephropathy

**DOI:** 10.1007/s11306-025-02371-8

**Published:** 2025-11-15

**Authors:** Yamilé López-Hernández, Juan José Oropeza-Valdez, Valeria Maeda-Gutiérrez, Jiamin Zheng, Rupasri Mandal, Juan Ernesto López-Ramos, José de la Cruz Moreira Hernández, Elena Jaime-Sánchez, María Fernanda Romo-García, José Antonio Enciso Moreno, David S. Wishart

**Affiliations:** 1 SECIHTI-Metabolomics and Proteomics Laboratory, Academic Unit of Biological Sciences, Autonomous University of Zacatecas, Zacatecas, Mexico; 2 Department of Biological Sciences, University of Alberta, Edmonton, AB T6G1C9 Canada; 3 Centro de Ciencias de la Complejidad, Universidad Nacional Autónoma de México (UNAM), Mexico City, Mexico; 4 Unidad Académica de Ingeniería Eléctrica, Universidad Autónoma de Zacatecas, Jardín Juárez 147, Centro, Zacatecas, Mexico; 5 Unidad Profesional Interdisciplinaria de Ingeniería Campus Zacatecas, Instituto Politécnico Nacional, Zacatecas, Mexico; 6 Hospital General de Zona No. 1, Dr Emilio Varela Luján, Instituto Mexicano del Seguro Social, Zacatecas, Zacatecas Mexico; 7 Área de Ciencias de La Salud, Campus UAZ Siglo XXI, Universidad Autónoma de Zacatecas, Zacatecas, Zacatecas Mexico; 8 Laboratorio de Inmunotoxicología, Unidad Académica de Ciencias Químicas, Universidad Autónoma de Zacatecas, Campus UAZ Siglo XXI, Zacatecas, Mexico; 9 Facultad de Química, Posgrado Química Clínica Diagnóstica, Universidad Autónoma de Querétaro, Santiago de Querétaro, Mexico

**Keywords:** Diabetic nephropathy, Diabetes, Targeted metabolomics, Metabolite, Urine, Biomarkers

## Abstract

**Introduction:**

Diabetic nephropathy (DN) is a major cause of chronic kidney disease and end-stage renal failure worldwide. The current diagnostic marker, albuminuria, lacks specificity and often detects renal damage only at advanced stages.

**Objectives:**

This study aimed to characterize urinary metabolic alterations associated with DN and explore metabolite panels with diagnostic potential.

**Methods:**

A targeted urinary metabolomics analysis was performed using the validated TMIC Urine MEGA Assay, quantifying 268 metabolites in 60 participants (20 controls, 20 type 2 diabetes mellitus [DM-2], and 20 DN patients). Data were analyzed by Partial Least Squares Discriminant Analysis (PLS-DA) for visualization, and penalized regression algorithms [Least Absolute Shrinkage and Selection Operator (LASSO) and Elastic Net (EN) with a Genetic Algorithm (GA)] followed by logistic regression (LR) modeling to identify potential discriminative variables.

**Results:**

DN patients showed marked alterations in metabolites related to oxidative stress, mitochondrial dysfunction, and inflammation. Twenty-four of 86 quantified uremic toxins differed significantly between DN and comparison groups. The LASSO-derived model identified β-alanine, kynurenine, glucose and argininic acid as key discriminants (AUC = 0.905, 10-fold CV), while inclusion of GFR and additional metabolites (2-hydroxybutyric acid, shikimic acid) improved performance (AUC = 0.96).

**Conclusions:**

Quantitative urinary metabolomics revealed metabolic perturbations reflective of DN pathophysiology and identified candidate metabolite panels with potential for non-invasive disease characterization. These findings, though preliminary, provide a foundation for validation in larger, longitudinal cohorts and for integrating urinary metabolomics into precision diagnostics for diabetic kidney disease.

**Supplementary Information:**

The online version contains supplementary material available at 10.1007/s11306-025-02371-8.

## Introduction

Diabetic nephropathy (DN), also known as diabetic kidney disease, is a serious microvascular complication of diabetes mellitus (DM) and a leading cause of chronic kidney disease (CKD) and end-stage renal disease (ESRD) (Alicic et al. 2017; de Boer 2011). Globally, 30–40% of diabetic patients develop DN (Sun 2022), characterized by a progressive decline in kidney function that, if inadequately managed, may necessitate dialysis or kidney transplantation in advanced stages. The prevalence of DN has been rising over time (Zhang 2016; Jha 2013).

DN progresses through distinct stages, beginning with microalbuminuria and a reduced estimated glomerular filtration rate (eGFR), progressing to persistent albuminuria, and ultimately culminating in ESRD (Thomas 2015; Disease 2011). While microalbuminuria is a conventional early biomarker for DN, it has limitations; structural kidney damage may precede microalbuminuria, and not all patients with microalbuminuria will progress to ESRD (Bakris and Molitch 2014). Hence, improved biomarkers are needed for early detection and monitoring of DN.

The development of DN is influenced by modifiable risk factors such as hypertension, glycemic control, dyslipidemia, and non-modifiable factors including age, genetics, and race (Bakris and Molitch 2014; Natesan and Kim 2021). Recent research has identified genetic markers like ACE, APOC1, and VEGF, which provide insights into the hereditary risk of DN (Natesan and Kim 2021; Song et al. 2023). Additionally, oxidative stress significantly contributes to DN pathogenesis; elevated glucose levels generate reactive oxygen species (ROS), leading to lipid peroxidation, DNA damage and inflammation (Yousef et al. 2023; Su et al. 2023; Wang and Zhang 2024; Aghadavod 2016; Lindblom et al. 2015). This oxidative environment encourages the accumulation of uremic toxins, such as asymmetric dimethylarginine (ADMA), which are known to impair renal function through endothelial dysfunction.

Metabolomics, through the comprehensive profiling of metabolites within biological samples, has emerged as a valuable tool for identifying early DN biomarkers (Zhang 2022; Luo et al. 2024). Urinary metabolomics, mainly, shows promise for measuring uremic toxins and other indicators of oxidative stress, mitochondrial dysfunction, and inflammation (André et al. 2023; Peris-Fernández 2024). The European Uremic Toxins (EUTox) Work Group has cataloged over 130 small molecule uremic toxins that accumulate in blood and urine due to reduced kidney function. This provides an essential framework for using metabolites to assess renal health (Vanholder et al. 2021).

To enhance feature discovery and mitigate overfitting in high-dimensional metabolomic data, we implemented different feature-selection strategies. Hybrid workflows combining methods such as Genetic Algorithms (GA) and Elastic Net (EN) regression have been shown to improve predictive subset identification in complex biological datasets (Amini and Hu 2021)and metabolomics applications (Tasci 2024). Our complementary pipeline (LASSO + GA/EN) builds upon benchmark evidence demonstrating that multi-algorithm strategies can outperform single-method approaches while acknowledging that, despite their promise, such models remain sensitive to sample size and cross-validation design. Within this analytical framework, we sought to comprehensively characterize the urinary metabolome across disease states. In this study we measured 268 urinary metabolites in healthy controls, type 2 diabetes mellitus (DM-2) patients, and DN patients. We used a newly developed assay, the TMIC Urine Mega Assay, which quantifies over 268 urinary metabolites including up to 40 acylcarnitines, over 60 amino acid-related compounds, nearly 60 organic acids, microbial metabolites, short-chain fatty acids, and more than 30 lipid compounds, including sphingomyelins, lysophospholipids, and phosphatidylcholines. Several metabolites included in this assay (up to 86) are classified as uremic toxins. We hypothesize that such an assay could detect unique metabolomic profiles in DN. These will reveal biomarkers linked to oxidative stress and immune dysregulation, shedding light on DN’s complex pathology and offering potential diagnostic markers for diagnosing and monitoring with future validation.

## Materials and methods

### Patient recruitment

Sixty subjects (20 per group) were recruited in Zacatecas, Mexico. Participants were recruited under a structured case–control design from the Endocrinology and Nephrology Department and the *DiabetIMSS* program of the Instituto Mexicano del Seguro Social (IMSS), which routinely monitors and follows up patients with diabetes mellitus and its complications, at the Instituto Mexicano del Seguro Social (IMSS). The DM-2 and DN groups were diagnosed under physician supervision following standard clinical and biochemical criteria, and age- and sex-matched controls without diabetes or renal dysfunction were recruited from the same clinical setting. Laboratory analyses were conducted for all patients to measure fasting glucose, glycated hemoglobin (HbA1c), creatinine, urea, general urine tests, 24-hour creatinine clearance (mL/min/1.72 m²), microalbuminuria, and urine proteins.

Inclusion criteria: Adult participants were classified according to glycemic status and renal function following the American Diabetes Association (ADA) and KDIGO guidelines (Committee 2024; Mogensen et al. 1983). Patients classified within the DN group were referred to the Nephrology Department and confirmed by a nephrologist. The Control (CTRL) group included subjects with normal HbA1c levels (< 5.6%) and normal renal function (eGFR ≥ 90 mL/min/1.73 m² and ACR < 30 mg/g). The DM-2 group comprised patients with HbA1c >6.5% and preserved renal function (GFR ≥ 90 mL/min/1.73 m² and ACR < 30 mg/g). The Diabetic Nephropathy (DN) group included patients with type 2 diabetes characterized by reduced GFR (< 60 mL/min/1.73 m²) and/or increased albuminuria (ACR > 300 mg/g). Patients monitored by the hospital service were classified after their renal function assessment according to the Mogensen and KDIGO classification (Mogensen et al. 1983; Stevens 2013), including laboratory results from blood chemistry, 24-hour urine protein (urine albumin levels/microalbuminuria), serum creatinine, and creatinine clearance. No imaging was used beyond routine clinical evaluation. Urine samples were collected under fasting conditions during routine clinical evaluation, immediately aliquoted, and stored at − 80 °C until analysis. Before processing, samples were thawed on ice, centrifuged at 3000 rpm to remove sediments, and the supernatant was directly applied to 96-well plates for metabolomic analysis. Exclusion criteria: patients with urinary tract infections or other renal pathologies. The study was approved by the IMSS National Committee for Scientific Research and Ethics (Registry R-2017-785-131) and adhered to the international ethical standards of the Helsinki Convention for research involving human subjects (World Medical Association 2013).

### Targeted metabolomics analysis

A custom-made liquid chromatography, direct flow injection tandem mass spectrometry (LC/DFI-MS/MS) assay (called the TMIC Urine MEGA) was used for the targeted identification and absolute quantification of up to 268 different endogenous metabolites in urine. These metabolites include amino acids and their derivatives, biogenic amines, acylcarnitines, glycerophospholipids, sphingomyelins, organic acids, nucleotide/nucleosides, among others. This assay is a modified version of the plasma MEGA assay, specifically adapted for urinary samples (Zhang 2024). A detailed description of the chemicals, standards, and internal standards (ISTD) preparation is provided in the Supplementary Material.

The assay was validated for urine samples (unpublished results). Quantification was done based on the peak area ratios of the targeted analyte compared to its isotope-labelled (ISTD). Calibration regression was built for each analyte within its concentration range, which was selected to fit normal human urinary concentrations. The Limit of Detection (LOD), Limit of Quantification (LOQ), Lower limit of Quantification (LLOQ), and Upper limit of Quantification (ULOQ) were determined for each metabolite. Evaluations of accuracy and precision were performed by analyzing Quality Control (QC) samples at three different concentration levels, with a formally defined criterion of accuracy being within 100 ± 20%, and precision within 20% acceptance criteria. The recoveries of absolutely quantified metabolites in pooled human urine at Low, Medium, and High concentration levels were also determined by comparing the calculated spiked concentration with the fortified amount (acceptable criteria of 20%).

#### LC/DFI-MS/MS analysis

Mass spectrometric analysis was performed on an ABSciex 5500 QTrap^®^ tandem mass spectrometer (Applied Biosystems/MDS Analytical Technologies, Foster City, CA) equipped with an Agilent 1290 series UHPLC system (Agilent Technologies, Palo Alto, CA). An Agilent reversed-phase Zorbax Eclipse XDB C18 column (3.0 mm × 100 mm, 3.5 μm particle size, 80 Å pore size) with a Phenomenex (Torrance, CA) A SecurityGuard C18 guard column (4.0 mm × 3.0 mm) was used for LC-MS/MS analysis. The controlling software for the sample analysis was Analyst 1.7.2 (Applied Biosystems/MDS Analytical Technologies, Foster City, CA). Data analysis was done using MultiQuant™ 3.0.3 (Applied Biosystems/MDS Analytical Technologies, Foster City, CA). Details of the MS method and parameters for the UHPLC system are provided in Supplementary Material.

### Statistical analysis

Baseline patient characteristics were summarized using medians with interquartile ranges (IQRs) or mean with standard deviations (s.d.) for continuous data and frequencies (%) for nominal data. Normality was evaluated with the D’Agostino-Pearson test. Continuous variables were compared using the Mann-Whitney U or Kruskal-Wallis tests, while nominal variables (e.g., sex, comorbidities) were analyzed using chi-square tests for trends. A p-value of < 0.05 was deemed statistically significant. Statistical analyses were performed using GraphPad Prism version 8.0.1 for Windows (GraphPad Software, La Jolla, CA, USA).

MetaboAnalyst 6.0 (Pang 2024) was employed for partial least squares discriminant analysis (PLS-DA) to explore urinary metabolomic data across study groups. Metabolites with more than 20% of missing values were excluded from further analysis. For the remaining metabolites, values below the limit of detection (LOD) were imputed using 1/5 of the minimum positive value of each variable (Left censored data estimation). Cross-validation (10-fold CV) and permutation tests (2000-fold) were applied to ensure the separation observed in PLS-DA was not due to random chance. Variable importance in projection (VIP) scores and heatmaps were generated, with significant features defined as those with a VIP score > 1.5 and a false discovery rate (FDR) < 0.05. The applied normalization encompassed normalization with creatinine, log transformation, and pareto scaling.

To identify metabolites discriminating between DM-2 and DN, two complementary strategies were applied: (1) penalized logistic regression with feature selection using the Least Absolute Shrinkage and Selection Operator (LASSO), followed by model refitting using standard logistic regression on the selected features; and (2) a Genetic Algorithm (GA) for feature selection coupled with EN–regularized logistic regression.

For the first strategy, metabolites with the highest LASSO coefficients (Supplementary Table S1) were used to construct a multivariate logistic regression model. Stepwise variable selection was subsequently applied to optimize model composition. To assess model robustness, a 10-fold cross-validation (CV) procedure was employed. Model performance was evaluated using the area under the receiver operating characteristic curve (ROC), sensitivity, and specificity. The final metabolite panel was selected based on the best overall logistic regression performance. Balanced sub-sampling–based Monte Carlo cross-validation (MCCV) was further used to generate ROC curves and estimate model variability. 95% confidence intervals (95% CIs) for AUC values were also computed. All analyses were performed using the MetaboAnalyst platform.

For the second strategy, a GA was employed for feature selection using binary-encoded individuals representing metabolite inclusion or exclusion. Each candidate subset was evaluated with an EN–regularized logistic regression model (L1/L2 ratio = 0.5; saga solver (sklearn.linear_model.LogisticRegression), with fitness quantified by the Matthews correlation coefficient (MCC) penalized for model complexity (> 8 features). The GA (DEAP v1.4.3) comprised 3,000 individuals evolved for up to 60 generations using two-point crossover (*p* = 0.8), adaptive bit-flip mutation (0.25 → 0.05), tournament selection (size = 4), and elitism (top 3 retained). A nested cross-validation framework ensured unbiased generalization: outer leave-one-out CV for evaluation and inner 4-fold stratified CV for GA optimization. Features selected in ≥ 50% of inner folds were used to retrain the final EN model and generate out-of-fold predictions. Model robustness was assessed via nonparametric bootstrap resampling (*n* = 2,000) to estimate 95% confidence intervals for AUC, accuracy, F1-score, sensitivity, specificity, MCC, and Brier score. All analyses were conducted in Python using scikit-learn v1.7.2.

## Results

### Clinical characteristics

Table 1 summarizes the clinical and biochemical characteristics of the study groups. Patients with DN were older, had higher weights, and exhibited a higher prevalence of hypertension compared to controls (*p* < 0.05). Biochemical parameters such as glucose, HbA1c, creatinine, and uric acid were significantly elevated in DN patients, while the glomerular filtration rate (GFR) was markedly lower (*p* < 0.05).

**Table 1:** Sociodemographic, epidemiological, and clinical characteristics of the participants by study group

Variable	CTRL	DM-2	DN	*P*-value
Sex (Female)	11 (55%)	11 (55%)	8 (40%)	ns
Age	46 ± 12	51 ± 5.8	57 ± 12.8	**
Hypertension	0 (0%)	9 (45%)	15 (75%)	***
Dyslipidemia	2 (10%)	7 (35%)	7 (35%)	ns
Alcohol	4 (20%)	7 (35%)	4 (20%)	ns
Duration of diabetes (years, median [IQR])	–	1.5 [0–3]	5 [2–12]	*
Insulin treatment (%)	–	10(50)	14(70)	*
Oral hypoglycemics (%)	–	11(55)	10(50)	ns
Antihypertensive treatment (%)	–	9(45)	15(75)	*
Lipid-lowering treatment (%)	2(10)	6(30)	7(35)	ns
Smoker	1 (5%)	4 (20%)	1 (5%)	ns
Weight (kg)	69.72 ± 13.66	78.22 ± 13.16	80.88 ± 12.05	*
Height (m)	1.60 ± 0.09	1.62 ± 0.08	1.66 ± 0.06	ns
BMI	27.15 ± 4.92	29.89 ± 5.36	29.64 ± 5.47	ns
Erythrocytes (million/µL)	4.83 ± 0.47	5.13 ± 0.48	4.80 ± 0.65	ns
Hemoglobin (g/dL)	14.42 ± 2.2	15.12 ± 1.37	14.26 ± 1.96	ns
Hematocrit (%)	42.94 ± 4.75	45.71 ± 3.53	42.87 ± 5.56	ns
Mean Corpuscular Volume (fL)	88.89 ± 4.89	89.42 ± 4.0	89.59 ± 5.45	ns
Hemoglobin Content (pg/cell)	29.77 ± 2.74	29.54 ± 1.59	29.8 ± 2.11	ns
Hemoglobin Conc. (%)	33.45 ± 1.94	33.05 ± 1.29	33.26 ± 1.1	ns
RDW (%)	13.49 ± 2.16	12.96 ± 0.73	13.38 ± 1.19	ns
Platelets (thousand/µL)	282.75 ± 78.08	261.05 ± 52.06	261.95 ± 63.9	ns
Leukocytes (thousand/µL)	6.23 ± 1.49	6.78 ± 1.42	7.17 ± 1.52	ns
Lymphocytes (thousand/µL)	2.08 ± 0.53	2.38 ± 0.55	2.01 ± 0.78	ns
Monocytes (thousand/µL)	0.43 ± 0.11	0.46 ± 0.08	0.46 ± 0.15	ns
Eosinophils (thousand/µL)	0.17 ± 0.14	0.17 ± 0.08	0.29 ± 0.17	ns
Basophils (thousand/µL)	0.04 ± 0.02	0.04 ± 0.02	0.03 ± 0.01	ns
Neutrophils (thousand/µL)	3.48 ± 1.14	3.71 ± 1.01	4.17 ± 1.27	ns
Immature Cells (thousand/µL)	0.03 ± 0.03	0.02 ± 0.01	0.02 ± 0.02	ns
Glucose (mg/dL)	85.92 ± 6.39	163.82 ± 65.51	128.53 ± 51.92	***
Creatinine (mg/dL)	0.73 ± 0.1	0.77 ± 0.15	1.38 ± 0.71	***
Uric Acid (mg/dL)	4.83 ± 1.31	4.88 ± 1.27	6.61 ± 1.73	***
HbA1c (%)	5.36 ± 0.27	8.72 ± 1.73	7.32 ± 1.65	***
Cholesterol (mg/dL)	176.66 ± 52.88	174.83 ± 61.77	185.63 ± 39.51	ns
Triglycerides (mg/dL)	143.03 ± 75.14	203.06 ± 107.87	229.50 ± 149.56	ns
HDL (mg/dL)	47.61 ± 8.92	40.89 ± 9.92	46.39 ± 13.86	ns
VLDL (mg/dL)	28.5 ± 12.76	40.75 ± 21.54	45.95 ± 29.88	ns
Glomerular filtration rate (GFR)	112.50 ± 14.28	107.80 ± 13.92	71.2 ± 31.95	***
Urine albumin-to-creatinine ratio (ACR)(mg/g)	16.65 ± 6.52	16.315 ± 8.27	380.085 ± 272.04	***

All variables are represented as the mean ± standard deviation for numerical variables and as count (%) for categorical variables. * Indicates *p* < 0.05, ** indicates *p* < 0.01, *** indicates *p* < 0.001, and ns indicates non-significant (*p* ≥ 0.05)

### Urine metabolomic profiling

#### Quantification and analytical validation

All urinary metabolites were quantified using the validated TMIC URINE MEGA Assay, a high-throughput targeted LC/DFI–MS/MS platform optimized for absolute quantification of small molecules in human urine. Analytical performance was verified according to international bioanalytical validation guidelines, with calibration curves exhibiting coefficients of determination (R² >0.99) and intra-/inter-assay precision within 15%. Recovery, linearity, and limits of detection and quantification (LOD/LOQ) were consistent with previously established TMIC assay standards. Quality control (QC) samples and ISTDs were included to monitor reproducibility and ensure data comparability across runs (unpublished results).

#### Urinary metabolic profiling

**Fig. 1:**
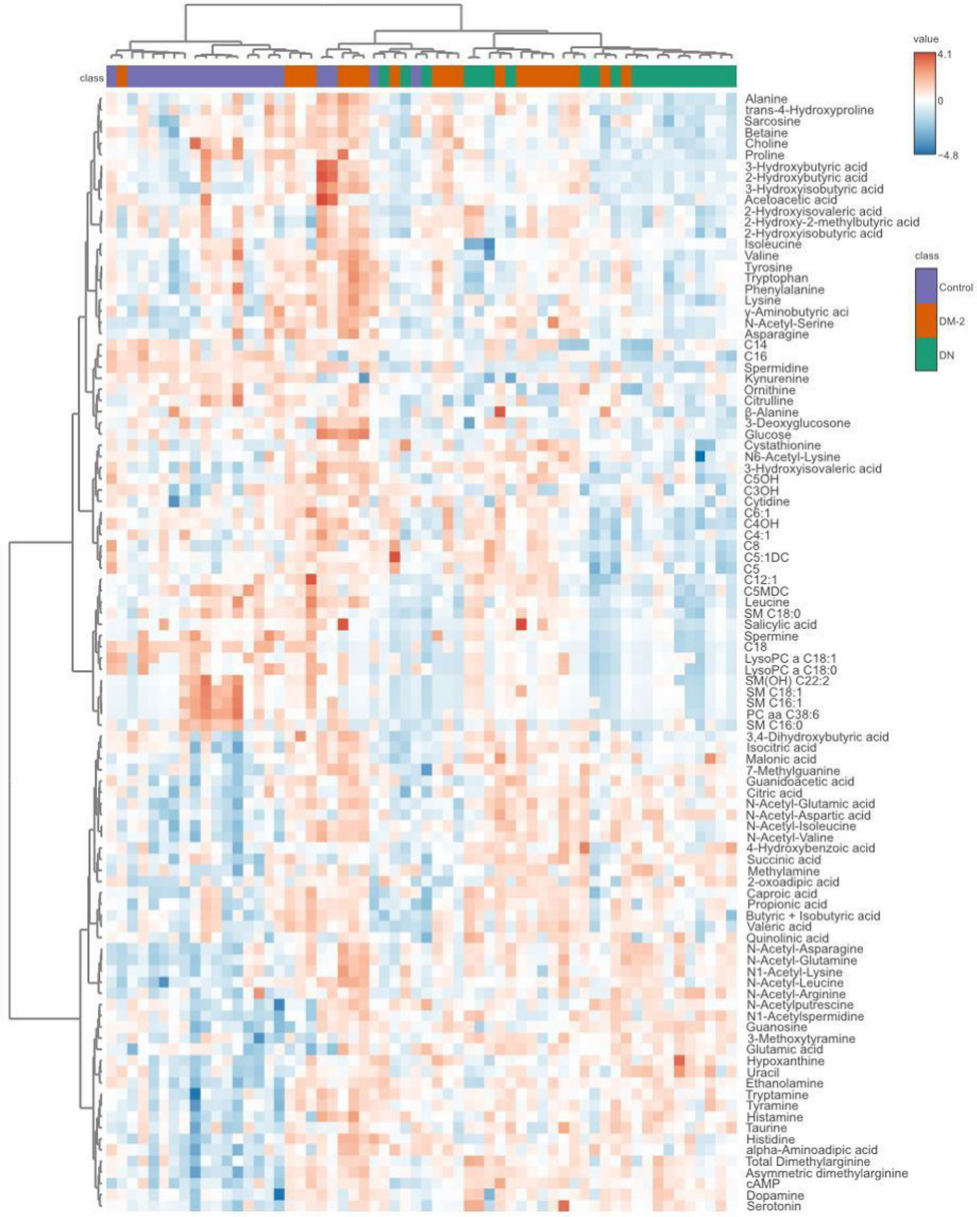
Urine Metabolomic Profiling: Heatmap of Metabolite Levels Across Study Groups. Heatmap of the top 100 metabolite levels across three study groups: Control, DM-2, and DN. The heatmap displays the relative abundance of metabolites, with color intensity indicating high (red) or low (blue) levels, as shown in the color scale. Hierarchical clustering, based on Pearson Distance

**Fig. 2:**
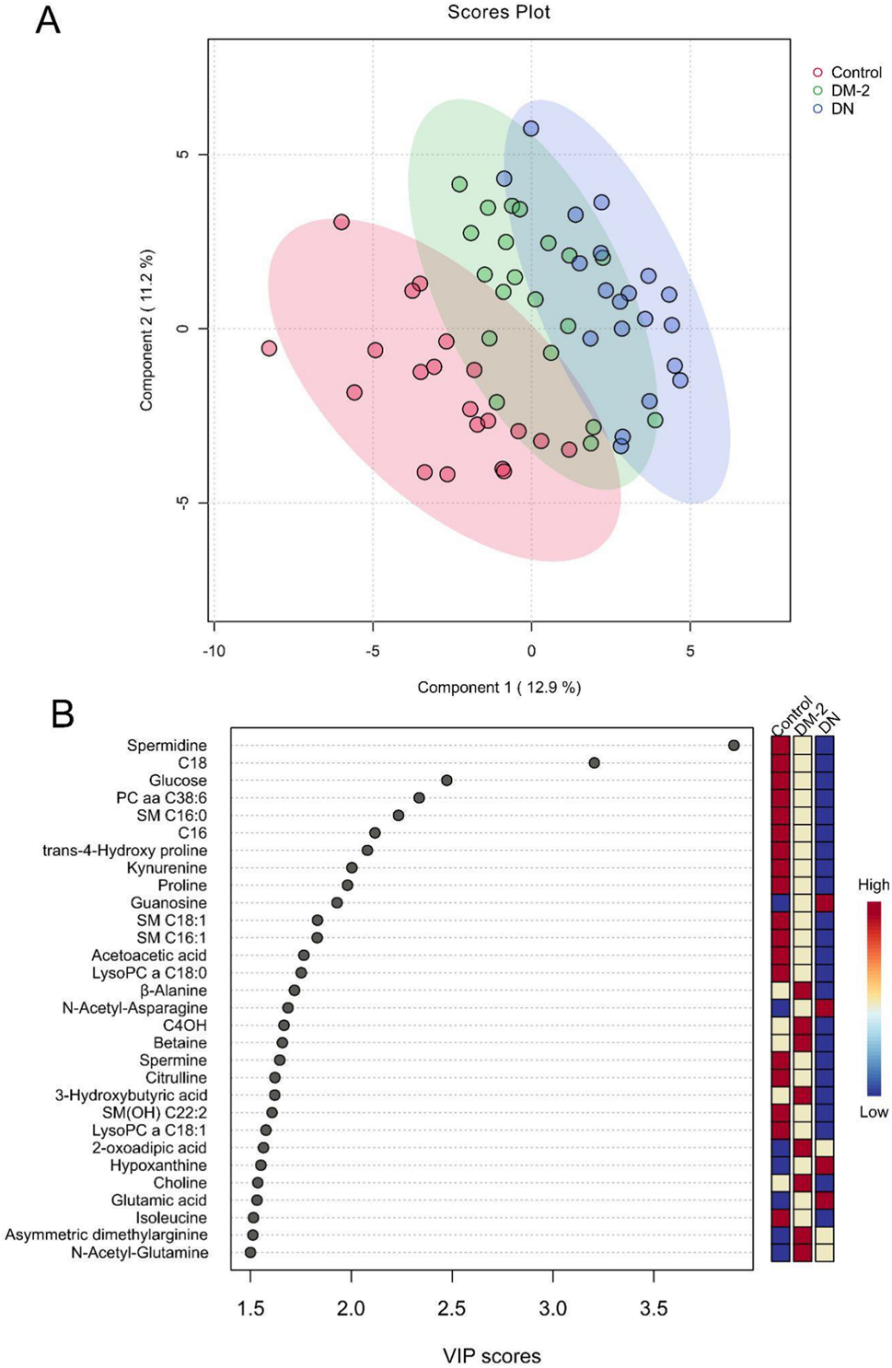
PLS-DA Analysis of Study Groups. Partial Least Squares Discriminant Analysis (PLS-DA) to differentiate the metabolic profiles of three study groups: control, DM-2, and DN. **A** shows a scores plot where each point represents an individual sample, color-coded by group (red for control, green for DM-2, and blue for DN), with ellipses indicating each group’s 95% confidence intervals. **B** Variable Importance in Projection (VIP) scores, identifying the top metabolites contributing to group discrimination, with higher VIP scores indicating greater importance. The color-coded bar next to the VIP scores shows the relative contribution of each metabolite across the groups

A hierarchical clustering heatmap (Fig. 1) revealed distinct metabolic patterns among the three study groups. Compared with controls, both DM-2 and DN participants exhibited progressively divergent profiles, suggesting cumulative metabolic perturbations as diabetes advances toward nephropathy. Partial Least Squares Discriminant Analysis (PLS-DA) confirmed clear group separation (Fig. 2A), and Variable Importance in Projection (VIP) scores identified the major contributors to this discrimination (Fig. 2B). Notably, spermidine, long-chain acylcarnitines (C18, C16), and several lysophosphatidylcholines (lysoPC a C18:0, lysoPC a C18:1) and sphingomyelins [SM(OH) C22:2, SM C16:1, SM C18:1, SM C16:0] were significantly decreased in DN, whereas short-chain acylcarnitine C4OH, guanosine, hypoxanthine, glutamic acid, and N-acetyl-asparagine were increased. The PLS-DA model demonstrated robust internal validation (Q² >0.5, *p* = 0.012 from 2000 permutations), confirming strong predictive capacity without evidence of overfitting (Supplementary Figures S1–S2).

### Accumulation of uremic toxins in DN patients

**Fig. 3:**
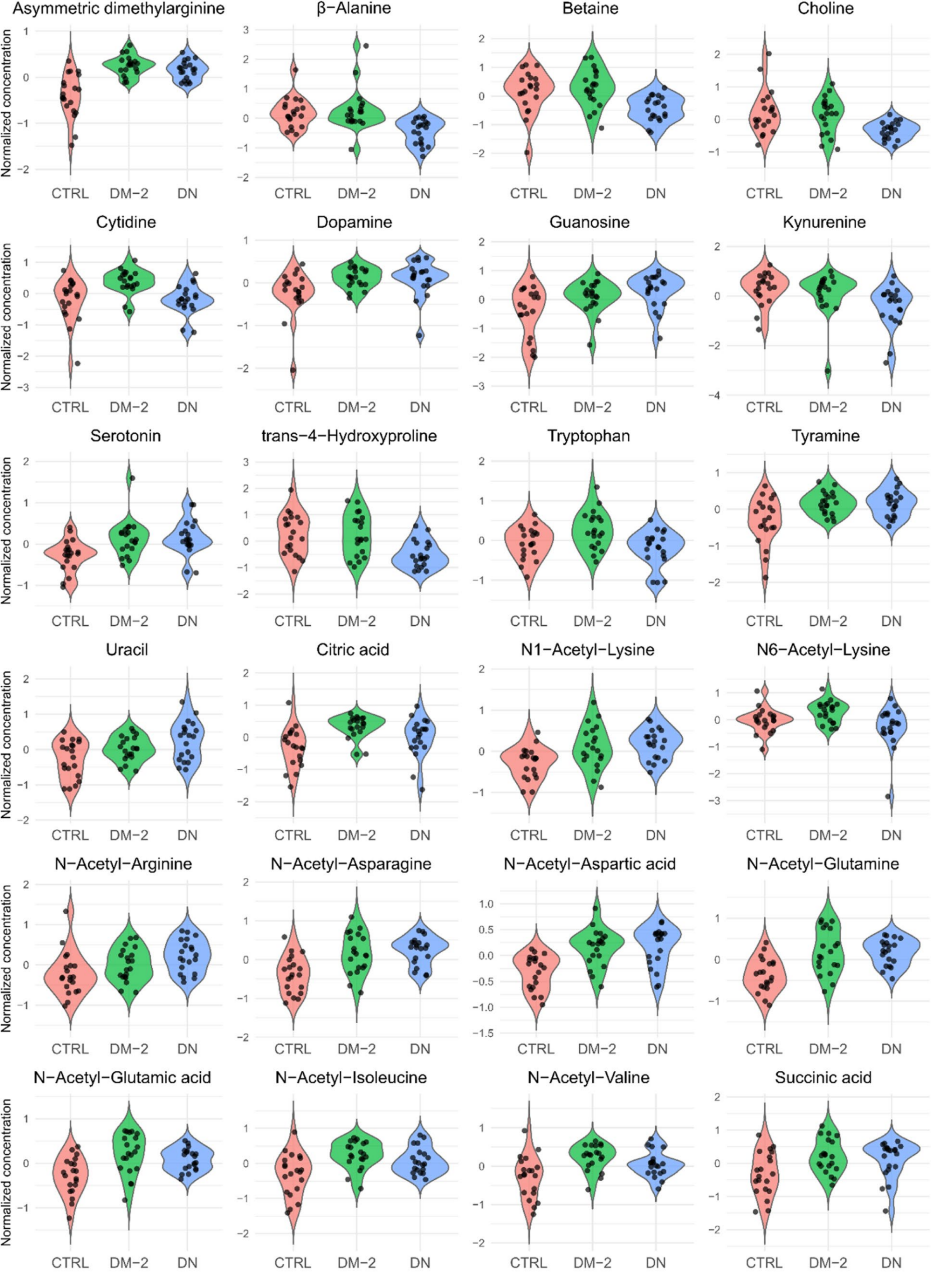
Violin dotplots of Uremic Toxins Across Study Groups. Violin dotplots comparing the normalized concentrations of various metabolites among three study groups: Red = CTRLs, Green = DM-2 and Blue = DN. Each violin represents the kernel density distribution of metabolite concentrations within each group, while individual dots correspond to single patient measurements. The width of each violin reflects the relative frequency of the data points, and the central black line indicates the median

A total of 86 small-molecule uremic toxins were measured. There were significant alterations in 24 of these uremic toxins in DN patients compared to CTRL and DM-2 patients (Supplementary Tables 2 and 3). Fourteen uremic toxins (ADMA, tyramine, serotonin, guanosine, uracil, succinic acid, dopamine, N-acetyl Asp, N-acetyl Gln, N-acetyl Asn, N-acetyl Glu, N-acetyl Val, N-acetyl Lys, and N-acetyl Arg) were elevated in DN patients compared to controls. However, no significant differences in uremic toxin levels were observed when DN patients were compared with DM-2 patients, except for uracil and N-acetyl arginine. Seven uremic toxins (β-alanine, cytidine, betaine, trans-hydroxyproline, choline, tryptophan, and kynurenine) were significantly decreased in the urine of DN patients compared to DM-2 patients (Fig. 3). Correlation analyses revealed negative associations between several uremic toxins and GFR; cytidine (*r* = −0.51, *p* < 0.05) and dopamine (*r* = −0.46, *p* < 0.05) exhibited significant negative correlations, while other uremic toxins demonstrated tendencies for inverse correlations (Supplementary Fig. 3).

### Metabolic differences between DM-2 and DN for disease diagnosis

**Fig. 4:**
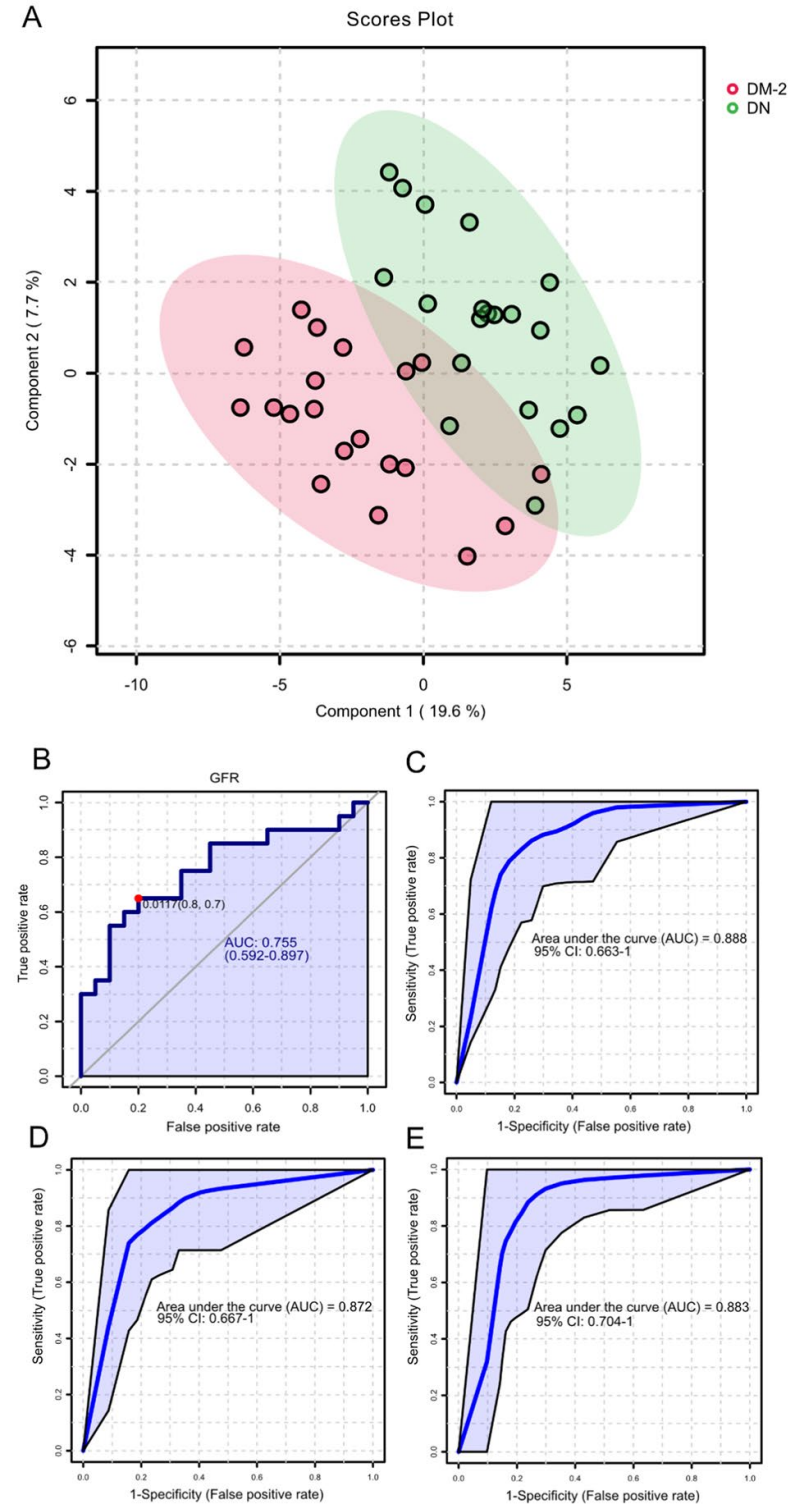
Urine Metabolic Differences Between DM-2 and DN Groups. **A** PLS-DA plot showing the differential clustering of DM-2 and DN metabolic profiles. **B** ROC curve of glomerular filtration rate (GFR) as a diagnostic indicator. **C** ROC curve of the metabolite-only model composed of β-alanine, kynurenine, and glucose. **D** ROC curve of the combined model integrating metabolites and GFR. **E** Alternative model composed of β-alanine, argininic acid, and kynurenine. AUC = area under the curve

The comparative analysis between DM-2 and DN groups revealed clear metabolic distinctions associated with nephropathy. Exploratory multivariate projection using PLS-DA (Fig. 4A) indicated distinct clustering between the two groups, reflecting global metabolic divergence. The main contributors to this separation included metabolites such as β-alanine, proline, betaine, glucose, kynurenine, and 2-hydroxybutyric acid.

When LASSO regression with cross-validation was applied (strategy 1), a sparse subset of metabolites that consistently contributed to group discrimination (based on penalized coefficients optimized for predictive performance) was selected. Metabolites that were repeatedly retained across cross-validated LASSO models and remained significant or near-significant in the refitted model were prioritized for inclusion in the final diagnostic panel. Following this criterion, β-alanine, kynurenine, and argininic acid were identified as the optimal predictors forming the final multivariate model used for validation. ROC analysis of GFR alone (Fig. 4B) confirmed its established role as a diagnostic indicator of DN (AUC = 0.755; 95% CI = 0.592–0.897). In contrast, the LASSO-derived metabolite panel composed of β-alanine, kynurenine, and glucose achieved markedly higher discriminative performance (Fig. 4C), with an AUC of 0.949 (95% CI: 0.928–0.969) in the training set and 0.905 (95% CI: 0.8–1.0) under internal 10-fold cross-validation. Incorporating GFR into this model (Fig. 4D) yielded an AUC of 0.883 (95% CI: 0.704–1), which did not significantly improve classification accuracy, indicating that the metabolite signature alone was sufficient for robust discrimination. However, because glucose levels can be influenced by antidiabetic medication, a complementary model excluding glucose was also evaluated to ensure biological and clinical validity (Fig. 4E). This alternative model, including β-alanine, argininic acid, and kynurenine, maintained strong predictive ability while minimizing potential confounding from treatment effects. In the training set, this model achieved an AUC of 0.949 (95% CI: 0.926–0.972). Under 10-fold cross-validation, it retained a high level of accuracy with an AUC of 0.886 (95% CI: 0.758–1.000). When GFR was added to this model, classification performance declined in the training set (AUC = 0.883; 95% CI = 0.704–1.000) and permutation testing showed that the improvement was not statistically significant (*p* = 0.062).

The final logistic regression equations were:

- Model 1: logit(P) = −0.975–6.644(β-Alanine) − 6.875(Glucose) − 8.125(Kynurenine) − 5.609(GFR).
- Model 2: logit(P) = −0.066–6.157(β-Alanine) + 3.052(Argininic acid) − 0.959(Kynurenine).

where, metabolite concentrations were creatinine-normalized, log-transformed, and Pareto-scaled.

The second strategy (EN-GA) successfully pinpointed a subset of metabolites consistently chosen across various cross-validation iterations, including β-alanine, argininic acid, 2-hydroxybutyric acid, and shikimic acid. Furthermore, EN logistic regression models, constructed using these GA-selected metabolites subsets, exhibited robust discriminative efficacy when assessed via LOOCV coupled with a bootstrap evaluation scheme.

**Fig. 5:**
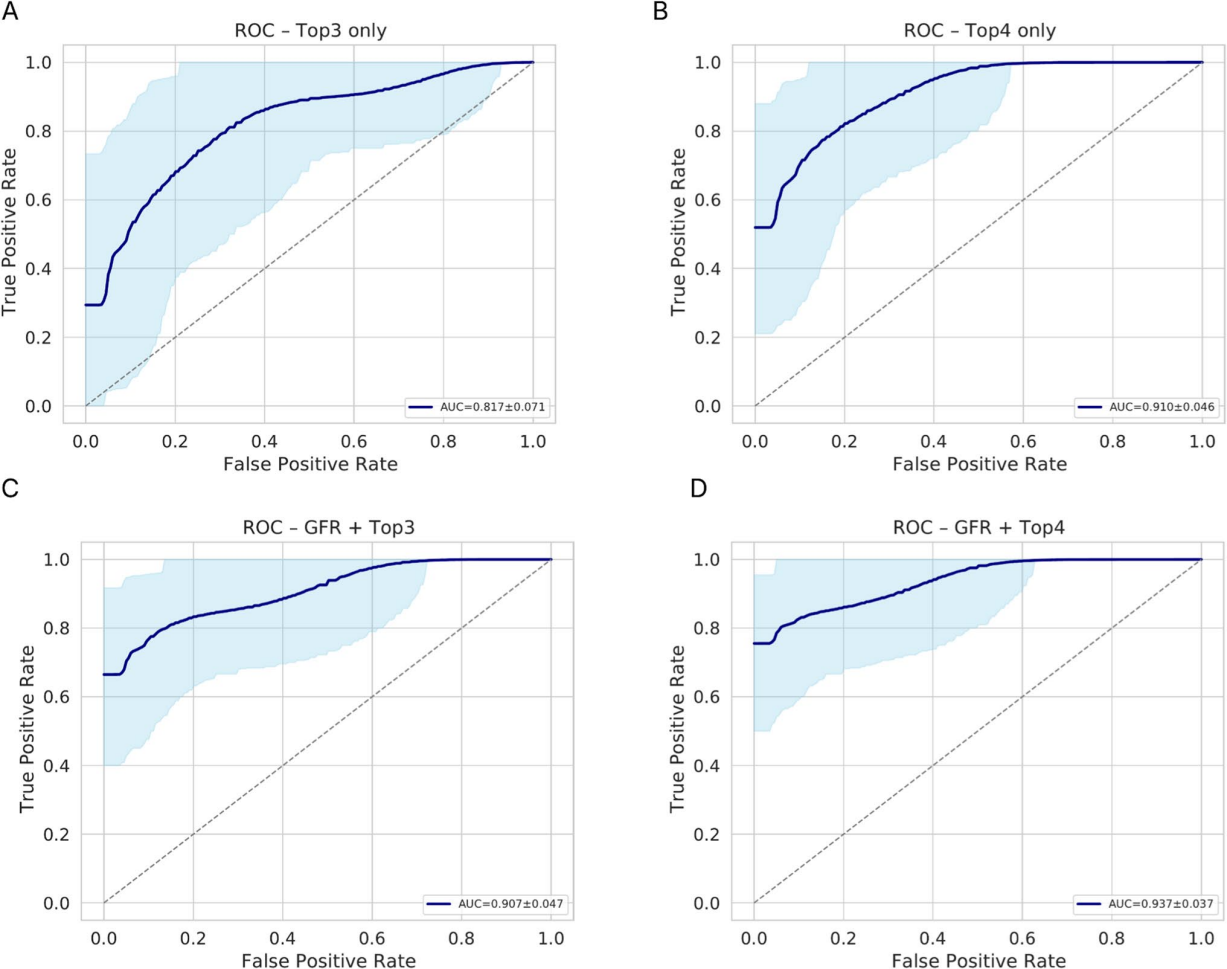
ROC curves for Elastic-Net logistic regression models using GA-selected metabolite subsets with and without GFR. **A** Top 3 metabolites: β-Alanine, Argininic acid, 2-Hydroxybutric acid. **B** Top 4 metabolites: β-Alanine, Argininic acid, 2-Hydroxybutric acid, Shikimic acid. **C** GFR + Top 3. **D** GFR + Top 4

As shown in Table 2; Fig. 5, panel A corresponds to the model including the top3 metabolites (β-Alanine, Argininic acid, 2-Hydroxybutyric acid), while panel B adds shikimic acid as the fourth feature (Top4). Panels C and D depict integration of GFR with the respective metabolite subset (GFR + Top3 and GFR + Top4). AUC values increased steadily, accompanied by consistent gains in accuracy, F1-score and MCC. The integration of GFR further strengthened classification performance, improving AUC by nearly 10% across all configurations and reducing bootstrap variability.

**Table 2:** Performance metrics of GA-selected metabolite subsets combined with GFR for DN classification

Model	AUC	ACC	F1	SENS	SPEC	MCC	BRIER
Top3 only	0.817 ± 0.07	0.749 ± 0.07	0.758 ± 0.08	0.800 ± 0.10	0.698 ± 0.10	0.502 ± 0.14	0.207 ± 0.01
GFR + Top3	0.907 ± 0.04	0.824 ± 0.06	0.816 ± 0.07	0.799 ± 0.09	0.849 ± 0.08	0.649 ± 0.12	0.150 ± 0.01
Top4 only	0.910 ± 0.04	0.774 ± 0.067	0.786 ± 0.071	0.849 ± 0.08	0.700 ± 0.10	0.555 ± 0.13	0.185 ± 0.01
GFR + Top4	0.937 ± 0.03	0.873 ± 0.05	0.860 ± 0.06	0.798 ± 0.09	0.949 ± 0.05	0.755 ± 0.09	0.138 ± 0.01

The ROC curves (Fig. 5) visualize this trend: models using metabolite subsets alone (panels A-B) showed moderate strong discrimination, while the inclusion of GFR (panels C-D) shifted the curves toward the upper-left quadrant with narrower confidence intervals denoting higher sensitivity and reduced false-positive rates.

### Pathway enrichment analysis reveals metabolic disruptions in DN

**Fig. 6:**
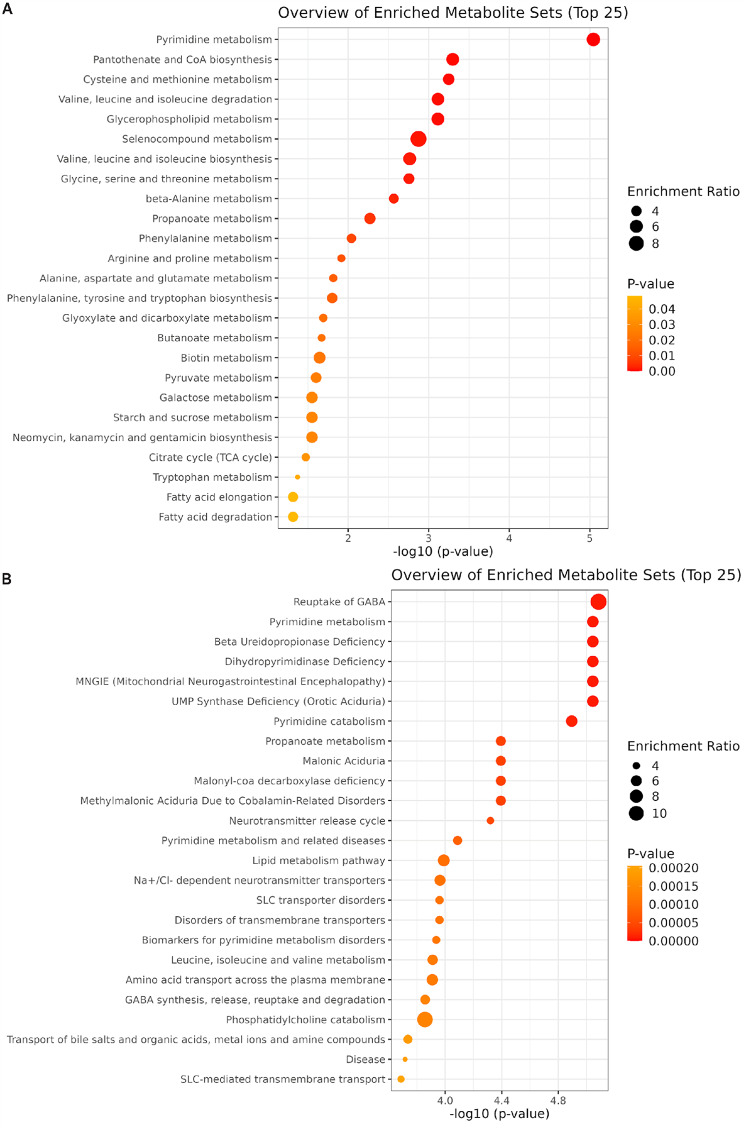
Bubble plots showing the top 25 significantly enriched metabolite sets from Over-Representation Analysis (ORA). **A** Results obtained using the RaMP-DB, illustrating pathways with high enrichment ratios (circle size) and significant p-values (color scale). **B** Results obtained using the KEGG database. The x-axis shows the negative log10 transformation of p-values, while circle size corresponds to the enrichment ratio

To further investigate the metabolic alterations distinguishing DM-2 from DN, we performed Over-Representation Analysis (ORA). The enriched pathways provided insights into the metabolic dysfunctions associated with DN, highlighting disturbances in amino acid metabolism, neurotransmitter signaling, mitochondrial function, and transport mechanisms (Fig. 5). Over-Representation Analysis using RaMP-DB (Fig. 6A) showed significant enrichment of pathways related to pyrimidine metabolism, including UMP synthase deficiency and beta-ureidopropionase deficiency, as well as GABA reuptake, propanoate metabolism, malonic and methylmalonic aciduria, and multiple transport-related processes (e.g., Na⁺/Cl⁻-dependent neurotransmitter transporters, SLC-mediated transmembrane transport). The highest -log10(p-value) values were observed for GABA reuptake and pyrimidine metabolism, with an enrichment ratio of up to 10 for these metabolic sets.

In parallel, Over-Representation Analysis conducted with the KEGG database (Fig. 6B) identified pyrimidine metabolism as the most enriched pathway, followed by pantothenate and CoA biosynthesis, cysteine and methionine metabolism, valine/leucine/isoleucine degradation and biosynthesis, and glycerophospholipid metabolism. Additional pathways include β-alanine metabolism, propanoate metabolism, and amino acid metabolism (phenylalanine, arginine, proline).

## Discussion

For metabolomics discoveries to progress beyond the exploratory stage and reach clinical implementation, it is critical that the detected metabolites are accurately identified and absolutely quantified. Establishing a list of potential diagnostic metabolites (particularly those already measured quantitatively) serves as a vital reference point for future studies, enabling researchers to build upon existing evidence rather than restarting from the discovery phase. Indeed, the most impactful advances in biomarker discovery, medical diagnostics, drug development, and environmental monitoring have been grounded in such targeted, quantitatively validated metabolite panels. This study underscores the significant advancements that quantitative urinary metabolomics could offer over traditional diagnostic models for DN. While conventional biomarkers, such as dipstick albuminuria and estimated GFR, remain widely used, their limitations include low specificity (AUC for GFR = 0.758, AUC for commonly detected albuminuria (≥ 300 mg/g) = 0.775), variability, and the need for early-stage detection, necessitating the exploration of more precise, non-invasive alternatives. Our study identified distinct metabolic signatures associated with DN diagnosis through targeted metabolomic analysis. The integration of metabolic profiling revealed a strong association between DN and alterations in pathways related to oxidative stress, mitochondrial dysfunction, and inflammation. However, pathway enrichment findings should be viewed as interpretive, reflecting downstream metabolic consequences of renal dysfunction rather than direct intracellular pathway dysregulation. Specifically, our study demonstrated significant perturbations in acylcarnitines, lysophosphatidylcholines, N-acetylated amino acids, and uremic toxins, contributing to renal function decline. Acylcarnitines are involved in fatty acid β-oxidation, and decreased levels in urine are related to renal function decline (Xia 2019). Lysophosphatidylcholines like lysoPC a C18:1 and lysoPC a C18:0 have been linked to inflammation and oxidative stress in kidney disease (Liu 2020). They can also be associated with atherosclerosis, a joint disease related to DM-2, and cardiovascular diseases (Paapstel 2018) with prevalence present in our sample space.

Several amino acids or amino acid metabolites were also altered. Most of them are considered uremic toxins. In fact, from our panel of uremic toxins (86 metabolites), about 30% resulted significantly altered in DN and DM-2 patients with respect to controls. In DN, urinary levels of N-acetylated amino acids may be altered due to renal dysfunction and metabolic disturbances. Some of the increased N-acetylated amino acids we found altered are also classified as uremic toxins. Damage to the proximal tubules impairs the reabsorption of these small molecules, resulting in their altered excretion in the urine. Oxidative stress, chronic inflammation, and heightened protein turnover in DN stimulate the overproduction of N-acetylated amino acids as part of the body’s response to cellular damage. This is further exacerbated by mitochondrial dysfunction, which disrupts energy metabolism and generates excess acetyl-CoA, fueling the acetylation process (Fan 2024; Chen et al. 2025). Acetylation serves as a detoxification mechanism, ensuring the neutralization of amino acids for excretion. Additionally, in early stages of DN, glomerular hyperfiltration may occur, increasing the passage of these metabolites into urine, while later stages see further disruption due to declining filtration efficiency. The changes in N-acetylated amino acids may indicate changes in protein acetylation, which are significant for regulating gene expression and enzyme activity (Vettore et al. 2021; Ree et al. 2018). Likewise, changes in these amino acids have been related to the development of CKD (Luo 2021). Similarly, a study conducted by Luo et al. (Luo 2021) found that higher circulating levels of five N-acetylated amino acids (N-δ-acetylornithine, N-acetyl-1-methylhistidine, N-acetyl-3-methylhistidine, N-acetylhistidine, and N2,N5-diacetylornithine) were associated with kidney failure.

**Fig. 7:**
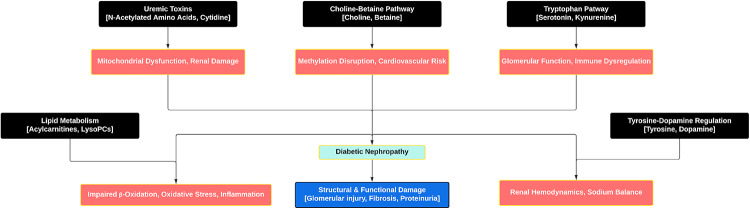
Schematic Diagram of Metabolic Pathways and Their Contributions to Diabetic Nephropathy Progression. Each black box at the top represents a dysregulated pathway or axis, lipid metabolism (acylcarnitines, lysophosphatidylcholines), uremic toxins (N-acetylated amino acids, cytidine), choline-betaine pathway (choline, betaine), tryptophan pathway (serotonin, kynurenine), and tyrosine-dopamine regulation (tyrosine, dopamine). Collectively, these disturbances lead to impaired fatty acid β-oxidation, oxidative stress, inflammation, mitochondrial dysfunction, altered methylation, immune dysregulation, and imbalanced renal hemodynamics. These interconnected pathogenic mechanisms ultimately affect DN, characterized by structural and functional kidney damage, including glomerular injury, fibrosis, and proteinuria

Among these uremic toxins, β-alanine, betaine, choline, glucose, argininic acid, and kynurenine were differentially expressed in DM-2 and DN patients. In diabetic nephropathy (DN), the urinary and plasma alterations of β-alanine, betaine, choline, and kynurenine reflect the metabolic progression from oxidative and osmotic stress in diabetes to tubular injury and inflammation. Reduced β-alanine and betaine indicate impaired tubular reabsorption and loss of osmoprotective capacity, while elevated choline and kynurenine suggest membrane breakdown, inflammatory activation, and oxidative stress (Zakrocka and Załuska 2022; Liu 2025). As renal function declines, the accumulation of uremic toxins further amplify damage through oxidative stress, endothelial dysfunction, inflammation, mitochondrial impairment, and fibrosis, perpetuating renal and systemic injury (Wojtaszek et al. 2021). Together, these uremic toxins delineate a biochemical continuum linking metabolic dysregulation to toxin retention and renal fibrosis, providing a mechanistic basis for identifying predictive urinary biomarkers in diabetic nephropathy (Fig.7).

Although similar studies have employed targeted metabolomics approaches on DN with serum samples (Chen 2025; Ibarra-González 2018; Zhang 2020), to the best of our knowledge, no quantitative metabolomic DN study has yet been performed on spot urine and no prior study of DN has been performed covering such a large number (268) of metabolites, allowing us to identify DN’s metabolic disruptions and potential biomarkers more comprehensively, offering insights into the complex metabolic network underlying DN.

To ensure robustness and minimize bias under small-sample, high-dimensional conditions, we implemented two complementary algorithms for feature selection and model construction. The first, LASSO logistic regression, performs embedded feature selection through L1 regularization, shrinking less informative coefficients to zero and thereby mitigating overfitting (Zou and Hastie 2005). The second, a GA coupled with EN logistic regression, combines an evolutionary search for optimal feature subsets with L1/L2 regularization to retain correlated predictors, a common property of metabolomic data where metabolites co-occur in biochemical pathways. The GA serves as a stochastic wrapper that explores nonlinear feature interactions and evaluates candidate subsets based on their aggregated predictive performance, thereby reducing redundancy compared to single-feature ranking. This dual-model framework enabled us to assess the stability and reproducibility of features across different regularization regimes. Concordant selection of metabolites by both approaches increases confidence that these variables reflect genuine biological structure rather than model-specific artifacts. Although the models achieved high internal AUC values, the limited sample size (*n* = 60) and possible metabolic overlap between advanced DM-2 and early DN require cautious interpretation pending external validation in larger, longitudinal cohorts. In our analyses, the EN model generally exhibited greater predictive stability and captured a more coherent metabolic signature than LASSO, though overall performance gains were modest. Both methods independently identified β-alanine and argininic acid as discriminant metabolites, underscoring their robustness across regularization frameworks and supporting their prioritization for replication and translational validation.

In contrast to traditional measures like albuminuria and GFR, which are well-established clinical indicators, several metabolites identified in this study demonstrated notable discriminative capacity between DM-2 and DN. The predictive models constructed demonstrated substantially higher AUC values compared to the commonly employed GFR (AUC of 0.758) and albuminuria commonly detected by dipsticks (Mejia 2021) (≥ 300 mg/g) (AUC of 0.775), which suggests their potential as superior, non-invasive markers. If validated in larger cohorts, a simple dipstick assay measuring these metabolites could potentially support or reduce reliance on invasive GFR tests, offering a more accessible and patient-friendly approach for diagnosing DN. Together, β-alanine, kynurenine, and glucose integrate metabolic, inflammatory, and glycemic pathways central to DN progression, supporting their joint inclusion in the diagnostic model. The inclusion of argininic acid, shikimic acid, and 2-hydroxybutyric acid in the discriminant metabolic panel highlights distinct yet convergent pathogenic axes in DN. Argininic acid reflects impaired arginine–NO balance and oxidative deamination consistent with endothelial dysfunction. Shikimic acid points to gut microbial dysbiosis and altered aromatic amino acid metabolism, supporting a gut–kidney metabolic link. Meanwhile, 2-hydroxybutyric acid captures oxidative stress and redox imbalance related to insulin resistance and glutathione turnover. Together, these metabolites integrate vascular, microbial, and redox pathways central to DN progression. While albuminuria and GFR remain central tools in clinical practice, our findings suggest that integrating the above metabolites could enhance the diagnosis and characterization of DN.

## Conclusion

This study highlights the potential of urinary metabolomics in the diagnosis and management of DN. The superior diagnostic accuracy of a small number of easily measured spot urine metabolites over traditional biomarkers, which require more invasive or onerous measures, reinforces the need for their potential inclusion or integration into clinical practice. This study establishes a quantitative urinary metabolomics framework for DN that, upon external validation, could complement GFR and albuminuria for early detection. Future work should expand cohort size, incorporate longitudinal follow-up, and assess cross-population reproducibility. Ultimately, leveraging metabolomics in routine clinical practice may improve DN management, enabling earlier detection, personalized treatment, and improved patient outcomes.

## Limitations

This study offers one of the first quantitative urinary metabolomic assessments of diabetic nephropathy (DN) using a fully validated targeted LC/DFI-MS/MS assay and integrative statistical modeling. The analytical workflow combined two complementary feature-selection strategies, LASSO regression and a Genetic Algorithm–EN framework, enhancing robustness against model bias and collinearity. The inclusion of absolute quantification for 268 metabolites, including 86 uremic toxins, provides a reproducible platform for future clinical validation. Nevertheless, several limitations warrant consideration. First, the modest sample size (*n* = 60; 20 per group) limits statistical power and increases uncertainty in effect-size estimates, particularly within multivariable models. Second, although clinically guided, the single-center, pilot case–control design may not capture population-level heterogeneity; future multicenter or longitudinal cohorts are needed to validate generalizability across the diabetes–nephropathy spectrum. Third, clinical covariates such as disease duration, pharmacologic treatment, and comorbidities were incompletely captured, introducing potential residual confounding. Fourth, despite employing penalized regression models and cross-validation to minimize overfitting, the limited number of observations per class can still inflate performance metrics (e.g., AUC-ROC). Finally, dietary, genetic, and environmental factors specific to the recruitment region could influence urinary metabolomic profiles. Accordingly, these findings should be regarded as preliminary and hypothesis-generating, providing a quantitative framework for larger, externally validated studies.

## Supplementary Information

Below is the link to the electronic supplementary material.

Supplementary Material 1

Supplementary Material 2

Supplementary Material 3

Supplementary Material 4

## Data Availability

The data reported in this study are accessible via Mendeley Data (https://data.mendeley.com/datasets/sv25hsx8vm/1).
