# Supplementary figures and images for: Comprehensive and quantitative urinary metabolomic profiling for improved characterization of diabetic nephropathy
Source: Metabolomics. 2025 Nov 15;21(6):163. doi: 10.1007/s11306-025-02371-8 (PMC12619831; doi:10.1007/s11306-025-02371-8)

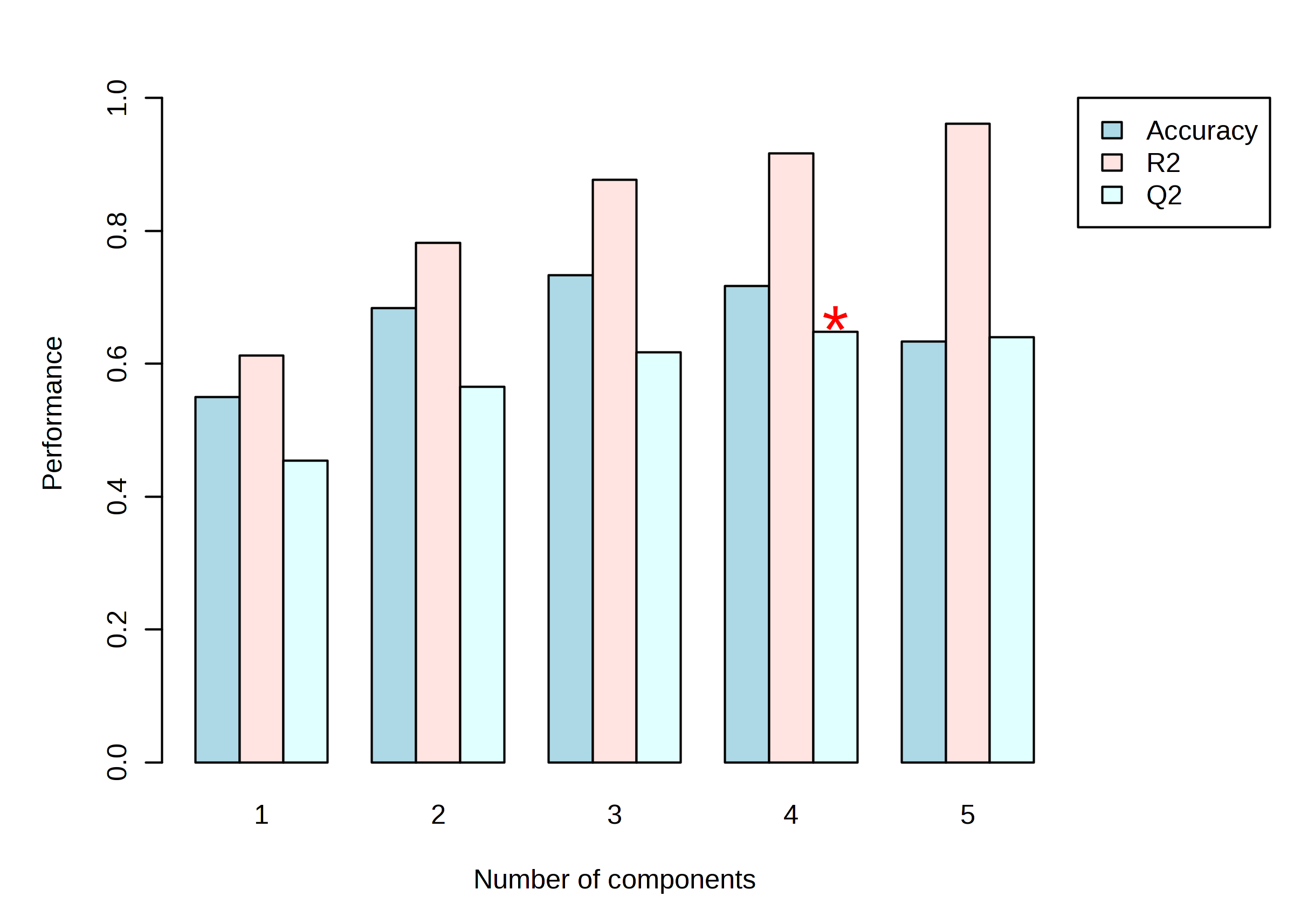

Supplement: Supplementary file 1 — Supplementary Material 1 [file 11306_2025_2371_MOESM1_ESM.png]

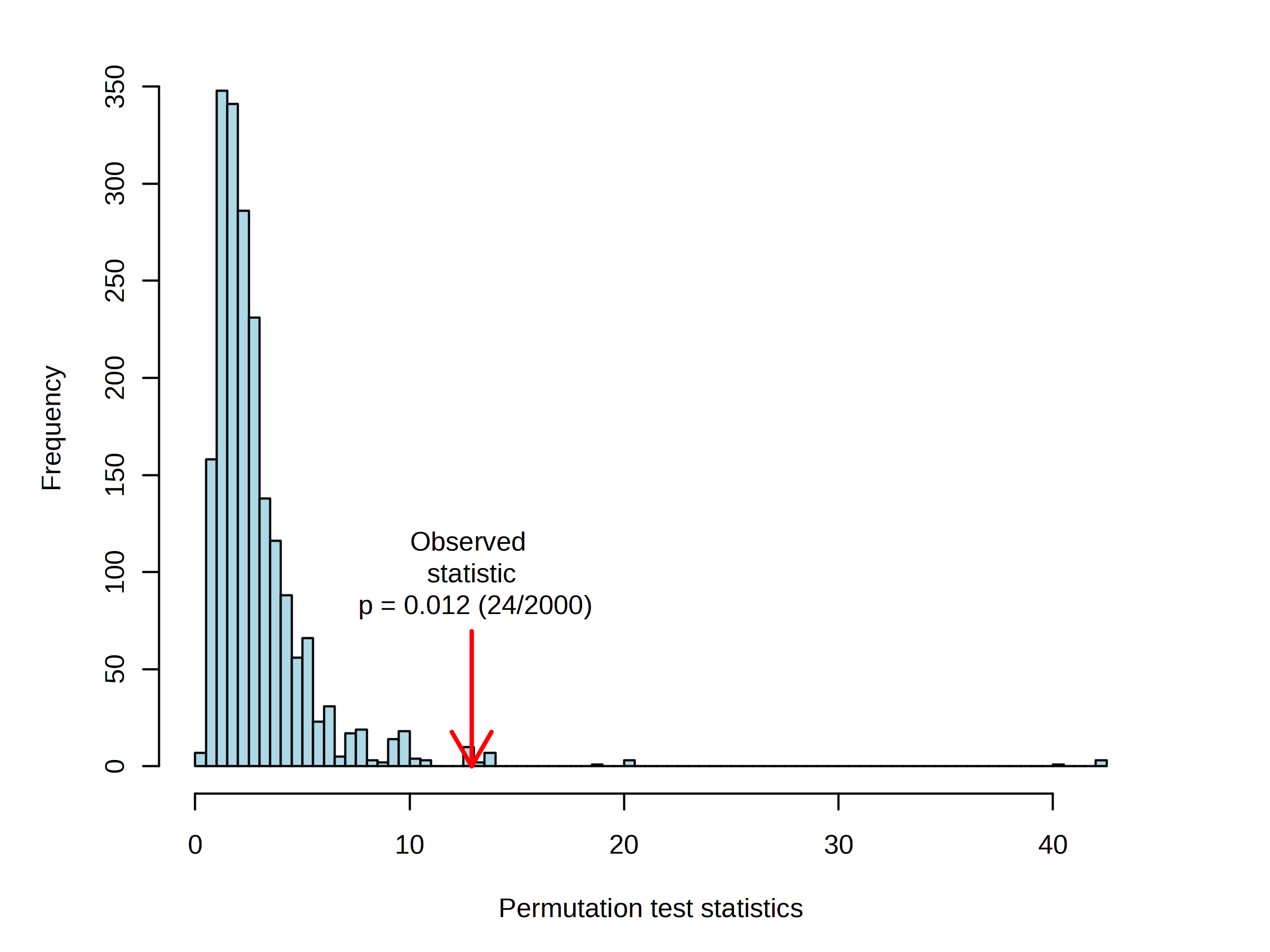

Supplement: Supplementary file 2 — Supplementary Material 2 [file 11306_2025_2371_MOESM2_ESM.png]

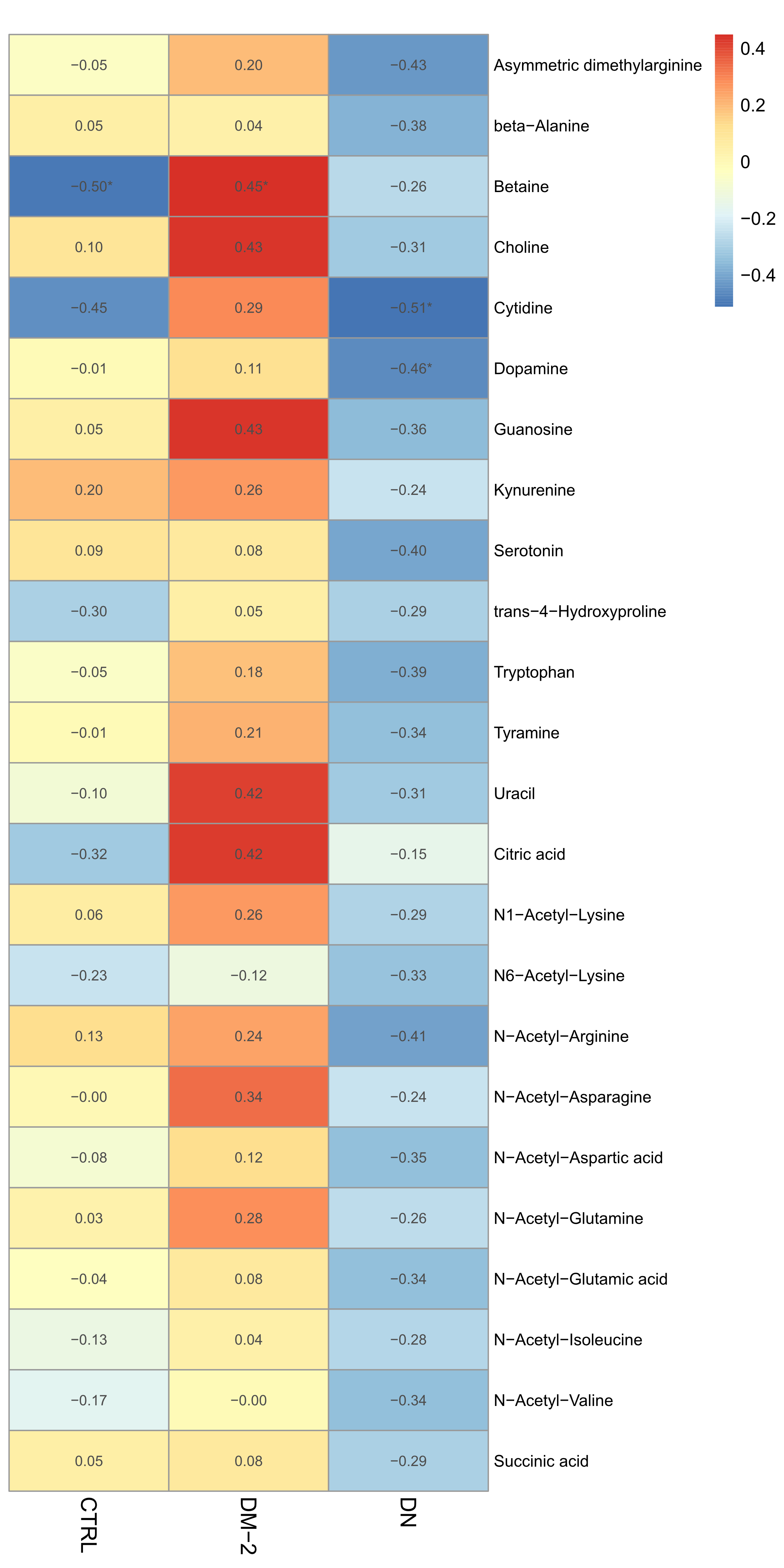

Supplement: Supplementary file 4 — Supplementary Material 4 [file 11306_2025_2371_MOESM4_ESM.png]
