# Supplementary material for: Comprehensive and quantitative urinary metabolomic profiling for improved characterization of diabetic nephropathy
Source: Metabolomics. 2025 Nov 15;21(6):163. doi: 10.1007/s11306-025-02371-8 (PMC12619831; doi:10.1007/s11306-025-02371-8)
Supplement: Supplementary file 3 — Supplementary Material 3 [file 11306_2025_2371_MOESM3_ESM.docx]

**Supplementary Information**

**
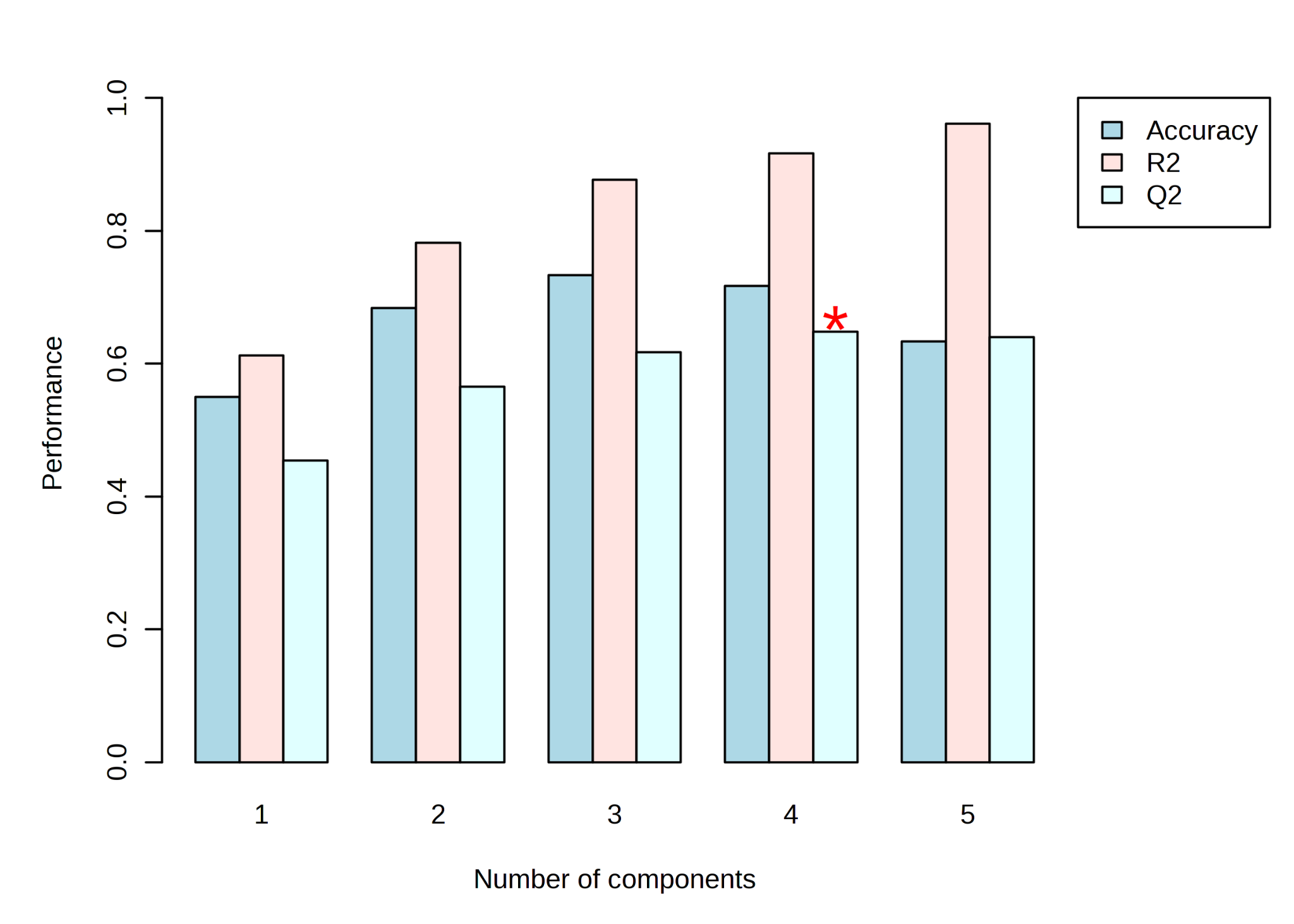
**

**Figure S1. Cross-validation performance of the PLS-DA model.** Bar plot showing model performance across 1–5 components in terms of classification accuracy (blue), R² (pink), and Q² (light blue).


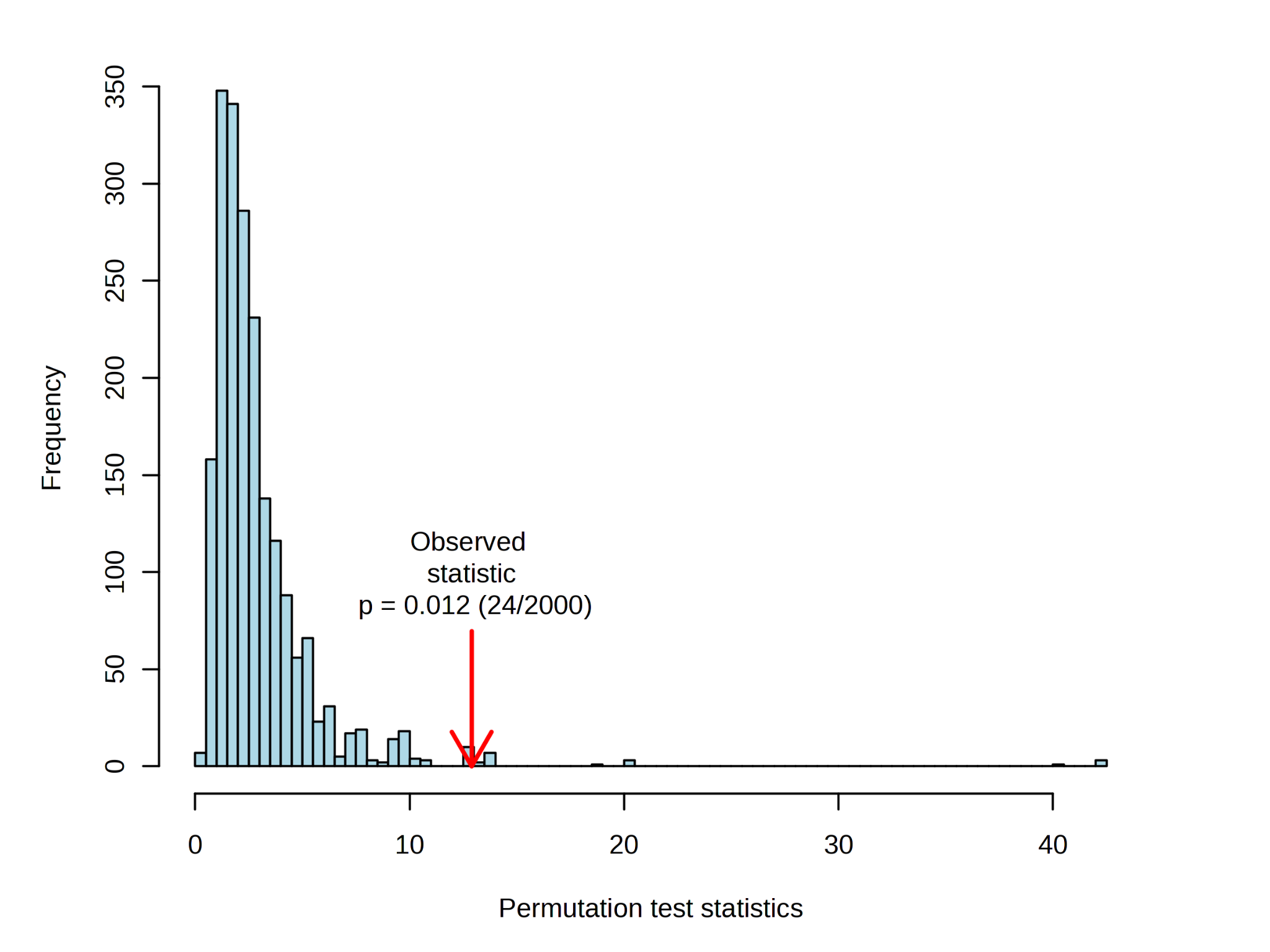


**Figure S2. Permutation test validating the PLS-DA model.** Distribution of test statistics from 2,000 permutations of class labels.


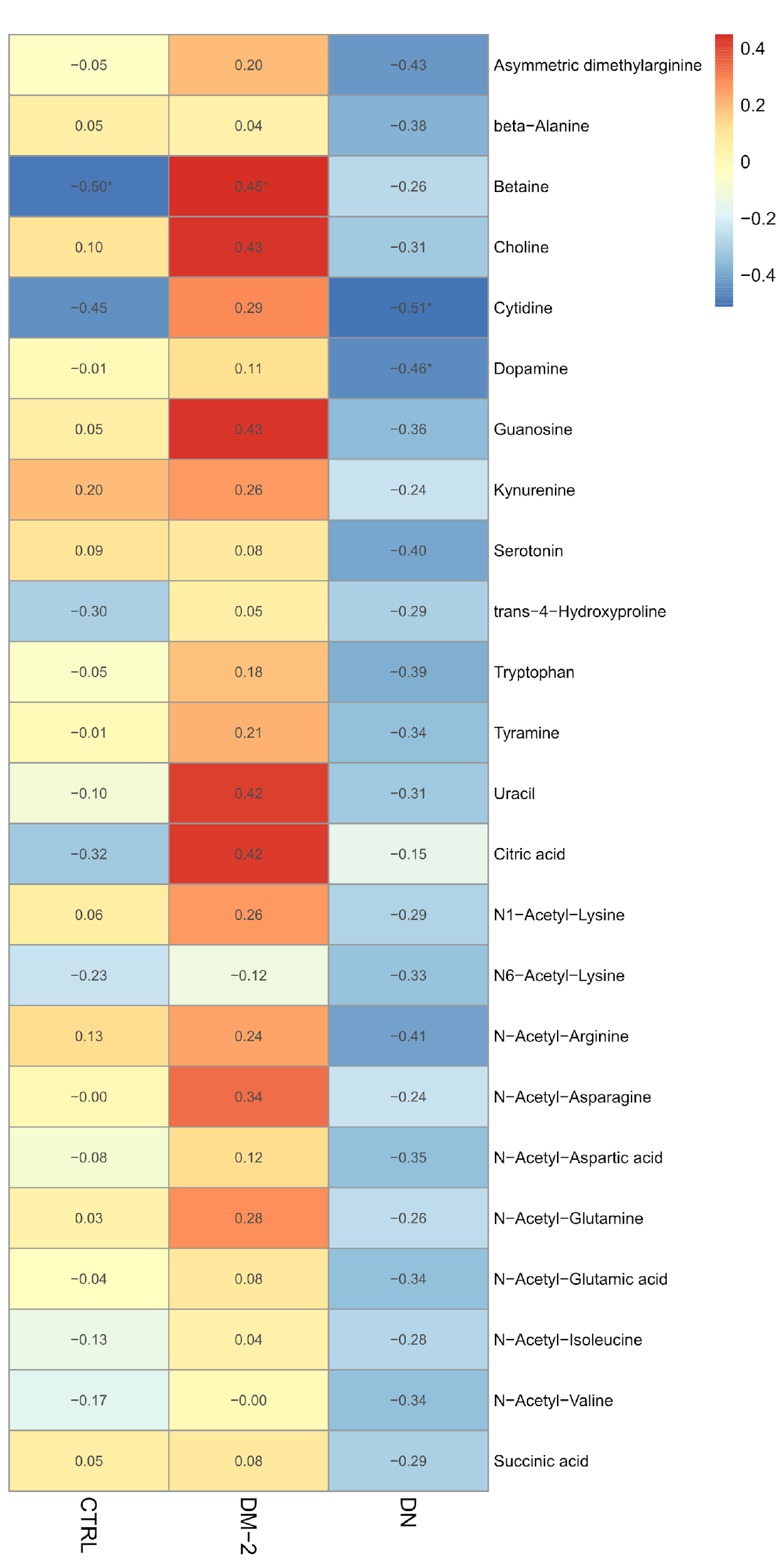


**Supplementary Figure 3. Correlation of Glomerular Filtration Rate (GFR) with Uremic Toxins**. Heatmap illustrating the correlation coefficients between glomerular filtration rate (GFR) and various uremic toxins across the three study groups: CTRL, DM-2, and DN. The color intensity and scale (ranging from -0.4 to 0.4) indicate the strength and direction of the correlations, with red representing positive correlations and blue representing negative correlations.

**Supplementary Table 1. Frequency of Metabolite Selection by LASSO Across Cross-Validation Iterations**
Summary of the most consistently selected metabolites during penalized logistic regression with LASSO.

| **Name** | **Lasso Freq (%)** |
| --- | --- |
| **β-Alanine** | 90.0 |
| **Shikimic acid** | 90.0 |
| **Valeric acid** | 90.0 |
| **Choline** | 80.0 |
| **Guanosine** | 80.0 |
| **C5:1DC** | 80.0 |
| **Argininic acid** | 80.0 |
| **cis-4-Hydroxyproline** | 70.0 |
| **Kynurenine** | 70.0 |
| **2-Hydroxybutyric acid** | 70.0 |
| **4-Ethylphenyl sulfate** | 70.0 |
| **5-Hydroxylysine** | 60.0 |
| **alpha-Aminobutyric acid** | 60.0 |
| **Glucose** | 60.0 |
| **3-Aminoisobutyric acid** | 60.0 |

**Supplementary Table 2. Uremic toxins dysregulated in DN patients.**

| **Metabolite** | **Comparison** | **CTRL** | **DM-2** | **DN** | **Direction** | **T test P.value** | **Anova P.value** |
| --- | --- | --- | --- | --- | --- | --- | --- |
| **Asymmetric dimethylarginine** | CTRL vs DM-2 | 2.06 ± 0.87 | 3.65 ± 0.79 |  | Up | *** | *** |
|  | CTRL vs DN | 2.06 ± 0.87 |  | 3.30 ± 0.67 | Up | *** |  |
|  | DM-2 vs DN |  | 3.65 ± 0.79 | 3.30 ± 0.67 | Down | ns |  |
| **β-Alanine** | CTRL vs DM-2 | 1.29 ± 1.60 | 2.40 ± 5.56 |  | Up | ns | *** |
|  | CTRL vs DN | 1.29 ± 1.60 |  | 0.43 ± 0.22 | Down | *** |  |
|  | DM-2 vs DN |  | 2.40 ± 5.56 | 0.43 ± 0.22 | Down | *** |  |
| **Betaine** | CTRL vs DM-2 | 36.53 ± 30.50 | 44.62 ± 43.63 |  | Up | ns | *** |
|  | CTRL vs DN | 36.53 ± 30.50 |  | 10.98 ± 6.93 | Down | ** |  |
|  | DM-2 vs DN |  | 44.62 ± 43.63 | 10.98 ± 6.93 | Down | *** |  |
| **Choline** | CTRL vs DM-2 | 1.64 ± 2.46 | 1.10 ± 0.75 |  | Up | ns | ** |
|  | CTRL vs DN | 1.64 ± 2.46 |  | 0.49 ± 0.17 | Down | ** |  |
|  | DM-2 vs DN |  | 1.10 ± 0.75 | 0.49 ± 0.17 | Down | ** |  |
| **Cytidine** | CTRL vs DM-2 | 0.03 ± 0.02 | 0.06 ± 0.03 |  | Up | ** | *** |
|  | CTRL vs DN | 0.03 ± 0.02 |  | 0.03 ± 0.02 | Up | ns |  |
|  | DM-2 vs DN |  | 0.06 ± 0.03 | 0.03 ± 0.02 | Down | *** |  |
| **Dopamine** | CTRL vs DM-2 | 0.11 ± 0.04 | 0.15 ± 0.03 |  | Up | * | * |
|  | CTRL vs DN | 0.11 ± 0.04 |  | 0.15 ± 0.05 | Up | * |  |
|  | DM-2 vs DN |  | 0.15 ± 0.03 | 0.15 ± 0.05 | Up | ns |  |
| **Guanosine** | CTRL vs DM-2 | 0.02 ± 0.02 | 0.04 ± 0.03 |  | Up | * | ** |
|  | CTRL vs DN | 0.02 ± 0.02 |  | 0.05 ± 0.03 | Up | ** |  |
|  | DM-2 vs DN |  | 0.04 ± 0.03 | 0.05 ± 0.03 | Up | ns |  |
| **Kynurenine** | CTRL vs DM-2 | 0.21 ± 0.19 | 0.16 ± 0.12 |  | Down | ns | * |
|  | CTRL vs DN | 0.21 ± 0.19 |  | 0.07 ± 0.08 | Down | ** |  |
|  | DM-2 vs DN |  | 0.16 ± 0.12 | 0.07 ± 0.08 | Down | * |  |
| **Serotonin** | CTRL vs DM-2 | 0.03 ± 0.01 | 0.05 ± 0.04 |  | Up | ** | ** |
|  | CTRL vs DN | 0.03 ± 0.01 |  | 0.05 ± 0.02 | Up | ** |  |
|  | DM-2 vs DN |  | 0.05 ± 0.04 | 0.05 ± 0.02 | Up | ns |  |
| **trans-4-Hydroxyproline** | CTRL vs DM-2 | 3.68 ± 6.62 | 3.31 ± 4.21 |  | Up | ns | ** |
|  | CTRL vs DN | 3.68 ± 6.62 |  | 0.58 ± 0.66 | Down | *** |  |
|  | DM-2 vs DN |  | 3.31 ± 4.21 | 0.58 ± 0.66 | Down | ** |  |
| **Tryptophan** | CTRL vs DM-2 | 3.55 ± 1.47 | 5.32 ± 3.10 |  | Up | * | ** |
|  | CTRL vs DN | 3.55 ± 1.47 |  | 3.11 ± 1.31 | Up | ns |  |
|  | DM-2 vs DN |  | 5.32 ± 3.10 | 3.11 ± 1.31 | Down | ** |  |
| **Tyramine** | CTRL vs DM-2 | 0.20 ± 0.12 | 0.30 ± 0.11 |  | Up | ** | ** |
|  | CTRL vs DN | 0.20 ± 0.12 |  | 0.30 ± 0.13 | Up | ** |  |
|  | DM-2 vs DN |  | 0.30 ± 0.11 | 0.30 ± 0.13 | Up | ns |  |
| **Uracil** | CTRL vs DM-2 | 1.49 ± 0.80 | 1.91 ± 0.77 |  | Up | ns | ** |
|  | CTRL vs DN | 1.49 ± 0.80 |  | 2.83 ± 1.93 | Up | ** |  |
|  | DM-2 vs DN |  | 1.91 ± 0.77 | 2.83 ± 1.93 | Up | ns |  |
| **Citric acid** | CTRL vs DM-2 | 177.27 ± 178.01 | 332.17 ± 120.89 |  | Up | *** | ** |
|  | CTRL vs DN | 177.27 ± 178.01 |  | 248.61 ± 167.00 | Up | ns |  |
|  | DM-2 vs DN |  | 332.17 ± 120.89 | 248.61 ± 167.00 | Up | * |  |
| **N1-Acetyl-Lysine** | CTRL vs DM-2 | 0.44 ± 0.18 | 0.72 ± 0.43 |  | Up | * | ** |
|  | CTRL vs DN | 0.44 ± 0.18 |  | 0.72 ± 0.27 | Up | *** |  |
|  | DM-2 vs DN |  | 0.72 ± 0.43 | 0.72 ± 0.27 | Up | ns |  |
| **N6-Acetyl-Lysine** | CTRL vs DM-2 | 0.39 ± 0.28 | 0.57 ± 0.32 |  | Up | * | * |
|  | CTRL vs DN | 0.39 ± 0.28 |  | 0.33 ± 0.23 | Up | ns |  |
|  | DM-2 vs DN |  | 0.57 ± 0.32 | 0.33 ± 0.23 | Down | ** |  |
| **N-Acetyl-Arginine** | CTRL vs DM-2 | 1.91 ± 1.74 | 2.19 ± 0.99 |  | Up | ns | * |
|  | CTRL vs DN | 1.91 ± 1.74 |  | 2.76 ± 1.20 | Up | ** |  |
|  | DM-2 vs DN |  | 2.19 ± 0.99 | 2.76 ± 1.20 | Up | ns |  |
| **N-Acetyl-Asparagine** | CTRL vs DM-2 | 2.27 ± 1.37 | 4.49 ± 2.74 |  | Up | ** | *** |
|  | CTRL vs DN | 2.27 ± 1.37 |  | 4.45 ± 1.73 | Up | *** |  |
|  | DM-2 vs DN |  | 4.49 ± 2.74 | 4.45 ± 1.73 | Up | ns |  |
| **N-Acetyl-Aspartic acid** | CTRL vs DM-2 | 2.39 ± 0.70 | 3.80 ± 1.30 |  | Up | *** | *** |
|  | CTRL vs DN | 2.39 ± 0.70 |  | 3.88 ± 1.25 | Up | *** |  |
|  | DM-2 vs DN |  | 3.80 ± 1.30 | 3.88 ± 1.25 | Up | ns |  |
| **N-Acetyl-Glutamine** | CTRL vs DM-2 | 1.27 ± 0.60 | 2.59 ± 1.51 |  | Up | *** | *** |
|  | CTRL vs DN | 1.27 ± 0.60 |  | 2.27 ± 0.75 | Up | *** |  |
|  | DM-2 vs DN |  | 2.59 ± 1.51 | 2.27 ± 0.75 | Up | ns |  |
| **N-Acetyl-Glutamic acid** | CTRL vs DM-2 | 1.23 ± 0.48 | 2.03 ± 0.79 |  | Up | *** | *** |
|  | CTRL vs DN | 1.23 ± 0.48 |  | 1.68 ± 0.41 | Up | ** |  |
|  | DM-2 vs DN |  | 2.03 ± 0.79 | 1.68 ± 0.41 | Down | ns |  |
| **N-Acetyl-Isoleucine** | CTRL vs DM-2 | 0.18 ± 0.13 | 0.30 ± 0.12 |  | Up | ** | ** |
|  | CTRL vs DN | 0.18 ± 0.13 |  | 0.24 ± 0.12 | Up | * |  |
|  | DM-2 vs DN |  | 0.30 ± 0.12 | 0.24 ± 0.12 | Down | ns |  |
| **N-Acetyl-Valine** | CTRL vs DM-2 | 0.28 ± 0.18 | 0.44 ± 0.13 |  | Up | ** | ** |
|  | CTRL vs DN | 0.28 ± 0.18 |  | 0.36 ± 0.14 | Up | * |  |
|  | DM-2 vs DN |  | 0.44 ± 0.13 | 0.36 ± 0.14 | Down | ns |  |
| **Succinic acid** | CTRL vs DM-2 | 5.39 ± 4.77 | 9.99 ± 7.30 |  | Up | ** | * |
|  | CTRL vs DN | 5.39 ± 4.77 |  | 8.35 ± 4.16 | Up | * |  |
|  | DM-2 vs DN |  | 9.99 ± 7.30 | 8.35 ± 4.16 | Up | ns |  |

All variables are represented as mean ± standard deviation (µmol metabolite/µmol creatinine). * Indicates p<0.05,** indicates p<0.01, *** indicates p<0.001, and ns indicates not significant (p ≥ 0.05). All statistical analyses,

including the determination of the "Direction," were performed using the normalized dataset, where metabolite

concentrations were creatinine-normalized, log-transformed, and Pareto-scaled. For each pairwise comparison, the

"Direction" reflects whether the mean value in the second group was higher ("Up") or lower ("Down") than that in

the first group.

**Supplementary Table 3. Uremic toxins measured in the study.**

| **Metabolite** | **LOD (μM)** | **Metabolite** | **LOD (μM)** |
| --- | --- | --- | --- |
| Adenine | 3.333 | Dimethylglycine | 0.093 |
| Adenosine | 1.113 | Fumaric acid | 0.301 |
| Asymmetric dimethylarginine | 0.612 | Glutaric acid | 0.088 |
| β-Alanine | 0.274 | Guanidinopropionic acid | 0.064 |
| Betaine | 17.924 | Hippuric acid | 0.128 |
| Choline | 0.127 | Homovanillic acid | 0.129 |
| Creatine | 0.012 | HPHPA | 0.009 |
| Creatinine | 0.151 | Indoxyl glucuronide | 0.067 |
| Cytidine | 0.035 | Indoxyl glucoside | 1.08 |
| Cytosine | 0.183 | Indoxyl sulfate | 1.287 |
| Diacetylspermine | 0.125 | Kynurenic acid | 0.011 |
| DOPA | 0.12 | Lactic acid | 2.783 |
| Dopamine | 0.013 | Methylmalonic acid | 0.057 |
| Guanine | 0.065 | N1-Acetyl-Lysine | 0.067 |
| Guanosine | 0.028 | N6-Acetyl-Lysine | 0.459 |
| Kynurenine | 0.002 | N-Acetyl-Alanine | 0.063 |
| Methylhistidine | 0.072 | N-Acetyl-Arginine | 0.057 |
| Nitro-Tyrosine | 0.609 | N-Acetyl-Asparagine | 0.219 |
| Phenylalanine | 0.702 | N-Acetyl-Aspartic acid | 0.115 |
| Sarcosine | 0.092 | N-Acetyl-Glutamine | 0.322 |
| Serotonin | 0.006 | N-Acetyl-Glutamic acid | 0.228 |
| Spermidine | 0.013 | N-Acetyl-Glycine | 0.198 |
| Spermine | 0.136 | N-Acetyl-Histidine | 0.17 |
| Thymidine | 0.528 | N-Acetyl-Leucine | 0.044 |
| Thymine | 0.273 | N-Acetyl-Methionine | 0.043 |
| Trimethylamine N-oxide | 0.01 | N-Acetyl-Proline | 0.051 |
| trans-4-Hydroxyproline | 0.131 | N-Acetyl-Serine | 0.098 |
| Tryptophan | 0.208 | N-Acetyl-Tryptophan | 0.379 |
| Tyramine | 0.016 | N-Acetyl-Tyrosine | 0.027 |
| Uracil | 0.24 | N-Acetyl-Valine | 0.124 |
| Uridine | 0.122 | Orotic acid | 0.176 |
| 2-hydroxyglutaric acid | 0.058 | p-Cresol sulfate | 2.557 |
| 3-Deoxyglucosone | 0.128 | Phenylacetic acid | 0.034 |
| 3-Hydroxybutyric acid | 0.167 | p-Hydroxyhippuric acid | 0.038 |
| 3-Indoleacetic acid | 16.2 | Propionic acid | 0.218 |
| 4-Ethylphenyl sulfate | 0.09 | Pyruvic acid | 0.179 |
| 4-Hydroxyphenylacetic acid | 0.311 | Quinaldic acid | 0.005 |
| 5-Hydroxyindoleacetic acid | 0.198 | Quinoline-4-carboxylic acid | 0.066 |
| alpha-Ketoglutaric acid | 0.197 | Quinolinic acid | 0.066 |
| Argininic acid | 0.151 | Succinic acid | 0.379 |
| Benzoic acid | 0.044 | Uric acid | 0.738 |
| Citric acid | 31.1 | Xanthine | 4.032 |
| CMPF | 0.01 | Xanthosine | 0.268 |

**Materials and Methods (Expanded)**

**Materials**

All chemicals used in this study were weighed individually on a Sartorius CPA225D semimicro electronic balance (Mississauga, ON, CA) with a precision of 0.0001 g. Stock solutions, with defined concentrations for each analyte, were prepared by dissolving the accurately weighed chemicals in proper solvents.

Chemical standards including acetylornithine, asymmetric dimethylarginine (ADMA), carnosine, creatinine, levodopa (DOPA), dopamine, histamine, methionine sulfoxide, cis-hydroxyproline, trans-hydroxyproline, phenylethylamine, putrescine, sarcosine, serotonin, spermidine, spermine, taurine, tyramine, alanine, arginine, asparagine, aspartic acid, citrulline, glutamine, glutamic acid, glycine, histidine, leucine, isoleucine, lysine, methionine, ornithine, phenylalanine, proline, serine, threonine, tryptophan, valine, 3-methylhistidine, betaine, trimethylamine N-oxide (TMAO), choline, nitro-tyrosine, tyrosine, kynurenine, creatine, alpha-aminoadipic acid, lactic acid, betahydroxybutyric acid, alpha-ketoglutaric acid, citric acid, butyric acid, isobutyric acid, propionic acid, succinic acid, fumaric acid, pyruvic acid, hippuric acid, methylmalonic acid, homovanillic acid, indole-3-acetic acid, uric acid, L-carnitine inner salt, acetyl-L-carnitine hydrochloride, propionyl-L-carnitine, butyryl-L-carnitine, hexanoyl-L-carnitine, octanoyl-L-carnitine, decanoylL-carnitine, dodecanoyl-L-carnitine, tetradecanoyl-L-carnitine, hexadecanoyl-L-carnitine, octadecanoyl-L-carnitine, N-stearoyl-D-erythro-sphingosylphosphorylcholine, 1,2-dilinolenoylsn-glycero-3-phosphocholine, 1,2-Dioctadecanoyl-sn-glycero-3-phosphocholine, 1-oleoyl-2hydroxy-sn-glycero-3-phosphocholine, and glucose were all bought from Sigma-Aldrich (Oakville, ON, CA). N^1^,N^12^ -diacetylspermine hydrochloride was purchased from Cayman Chemical (Ann Arbor, MI, U.S.A.). 4-hydroxyhippuric acid was obtained from Toronto Research Chemicals (North York, ON, CA). Other stable isotope (D, ^13^ C, ^15^ N)-labelled internal standards (ISTD) including D _2_ -ornithine, N^15^-histidine, D _3_ -creatinine, D _3_ -DOPA, D _4_ -dopamine, ^13^C-tyrosine, ^13^ C-D_3_ -methionine, D_3_ -proline, D _4_ -serotonin, D _4_ -putrescine, D _3_ -sarcosine, ^13^ C _2_ -taurine, D _4_ -tyramine, ^15^N-alanine, ^13^C _6_ -arginine, ^15^N-asparagine, D _3_ -aspartic acid, D _7_ -citrulline, D _3_-glutamic acid, D _5_ -glutamine, ^13^C _2_ -glycine, ^13^C-leucine, ^15^N-phenylalanine, ^13^C-serine, D _2_- threonine, ^15^ N _2_ -tryptophan, D _8_ -valine, D _9_ -TMAO and ^15^ N _2_ -uric acid were bought from Cambridge Isotope Laboratories Inc. (Tewksbury, MA, U.S.A.). D _8_ -spermine tetrahydrochloride, D _8_ -spermidine trihydrochloride were purchased from IsoSciences (Ambler, PA, U.S.A.). D _6_ -N ^1^, N ^12^ diacetylspermine dihydrochloride and D^6^ -ADMA were bought from Toronto Research Chemicals (North York, ON, CA). D _3_ -creatinine, D _9_ -choline chloride, D _9_ -betaine hydrochloride, 2-D _1_ sodium L-lactate, D _4_ -sodium beta-hydroxybutyrate, 1- ^13^ C-alpha-ketoglutaric acid, D _4_ -citric acid, 1- ^13^ C-butyric acid, 1- ^13^ C-propionic acid, D _4_ -succinic acid, ^13^ C _2_ -D _2_ -fumaric acid, ^13^ C-pyruvic acid, D _2_ -hippuric acid, methyl-D _3_ -malonic acid, and D _2_ -indole-3-acetic acid were purchased from C/D/N Isotopes Inc. (Pointe-Claire, QC, CA). Labelled carnitine standard set was purchased from Cambridge Isotope Laboratories Inc. (Tewksbury, MA, U.S.A.). 1,2-dimyristoyl-sn-glycero-3phosphocholine, 1,2-diarachidoyl-sn-glycero-3-phosphocholine, 1-nonanoyl-2-hydroxy-snglycero-3-phosphocholine, N-hexanoyl-D-erythro-sphingosylphosphorylcholine, and ^13^ C_6_ glucose were bought from Sigma-Aldrich (Oakville, ON, CA).

For amino acids, amino acid derivatives, biogenic amines, and nucleotide/nucleosides, OptimaTM-LC-MS-grade water (Fisher Scientific, Ottawa, ON, Canada) was used as the solvent for preparing stock solutions, calibration mixtures, QC mixtures, and a working ISTD solution mixture. The working ISTD solution mixture with defined concentrations of ISTDs in LC/MS-grade water was prepared by mixing all the isotope-labeled stock solutions. Chloroform (Sigma-Aldrich, Oakville, ON, Canada) was used for preparing stock solutions for lipids. Methanol was used for preparing acylcarnitines and hexose stock solutions. For lipids, acylcarnitines, and hexose, a working calibration mixture with defined concentrations of standards and a working ISTD solution mixture with defined concentrations of ISTDs in LC/MS-grade methanol were also made by mixing all the prepared stock solutions.

For organic acids, 75% (*v*/*v*) OptimaTM-LC-MS-grade methanol (Fisher Scientific, Ottawa, ON, Canada) in OptimaTM-LC-MS-grade water was used for preparing stock solutions and calibration mixtures. An ISTD solution mixture with standards having defined concentrations was prepared in the same way as the calibration standards. A working internal standard solution mixture was prepared by derivatizing the ISTD solution with an isotope-labeled chemical derivatization reagent (^13^C6-3-nitrophenylhydrazine or 3-NPH) during the sample preparation procedure.

**Methods**

PITC derivatization was used for amino acids, amino acid derivatives, and biogenic amines, and nucleotide/nucleosides (Panel A). This panel uses a 96-deep-well plate (NuncTM 96 DeepWell plate, Fisher Scientific, Ottawa, ON, Canada) with a 96-well filter plate (Multiscreen “solvinert” filter plates, hydrophobic, PTFE, 0.45 μm, clear, nonsterile, Fisher Scientific, Ottawa, ON, Canada) attached via sealing tape, and a set of reagents and solvents used to prepare the plate assay. To each urine sample (or QC standard), the ISTD mixture solutions were pipetted directly onto the center of each corresponding spot/well in the upper filter plate. After drying the plate under a stream of nitrogen for 30 min, 50 μL of the 5% PITC derivatization solution (where 300 μL of PITC reagent (Sigma-Aldrich, Oakville, ON, Canada) was added to a mixture of ethanol (Fisher Scientific Ottawa, ON, Canada), LC/MS water, and pyridine (Sigma-Aldrich, Oakville, ON, Canada), each 1900 μL) was added to each well. The reaction was kept at room temperature for 20 min, followed by another 1.5 h drying under a gentle nitrogen stream to remove the excess PITC solution. To extract the targeted analytes, 300 μL of LC/MS-grade methanol containing 5 mM ammonium acetate (Fisher Scientific Ottawa, ON, Canada) was then added to each spot. The whole plate was covered and shaken at 300 rpm for 30 min at room temperature, and then centrifuged at 50× *g* for 5 min to collect the extracts from the upper filter plate to the bottom collection plate. Finally, 50 μL of extracts were transferred to a new 96-deep-well plate and then diluted with 450 μL of LC/MS-grade water for LC-MS/MS analysis to quantify amino acids, amino acid derivatives, biogenic amines, and nucleotide/nucleosides. Moreover, 10 μL of the remaining extracts were transferred to another new 96-deep-well plate and then diluted with 490 μL of DFI buffer for direct flow injection-tandem mass spectrometry (DFI-MS/MS) analysis. This was conducted to quantify the lipids, acylcarnitines, and glucose/hexose (which are not derivatized by PITC). The assay was completed to quantify the lipids, acylcarnitines, and glucose/hexose.

For organic acid analysis of the urine samples, 50 µL of each sample was directly loaded into a 96-well plate (Panel B). Then, 75 µL of derivatization reagent (which consisted of 25 µL of 250 mM 3-NPH (Sigma-Aldrich, Oakville, ON, Canada) in 50% aqueous methanol, 25 µL of 150 mM 1-ethyl-3-(3-(dimethylamino)propyl) carbodiimide (EDC, Sigma-Aldrich, Oakville, ON, Canada) in methanol, and 25 µL of 7.5% pyridine in 75% aqueous methanol) was added to each well. In a 1.5-mL Eppendorf tube, 125 µL of the working ISTD solution was prepared by mixing 50 µL of the ISTD mixture solution with 75 µL of isotope-labelled derivatization reagent (which consisted of 25 µL of 250 mM ^13^C6-3-NPH (Cayman chemical, Ann Arbor, MI, USA) in 50% aqueous methanol, 25 µL of 150 mM 1-ethyl-3-(3-(dimethylamino)propyl) carbodiimide (EDC) in methanol, and 25 µL of 7.5% pyridine in 75% aqueous methanol). The 96-well plate was then shaken at 500 rpm for 2 h at room temperature, followed by adding 325 µL of LC/MS water and 50 µL of butylated hydroxytoluene (BHT) dissolved in methanol (2 mg/mL) to each well of the plate. The working ISTD solution was shaken along with the plate under the same conditions for 2 h and then diluted with 1125 µL of LC/MS water. Moreover, 10 µL of the diluted working ISTD solution was then loaded to each well of a new deep well plate except for the double blank sample position, followed by transferring 25 µL of the derivatized samples to the corresponding wells of the new plate.

**LC/DFI-MS/MS Analysis**

Mass spectrometric analysis was performed on an ABSciex 5500 QTrap^®^ tandem mass (MS/MS) spectrometer (Applied Biosystems/MDS Analytical Technologies, Foster City, CA, USA) equipped with an Agilent 1290 series UHPLC system (Agilent Technologies, Palo Alto, CA, USA). An Agilent reversed-phase Zorbax Eclipse XDB C18 column (3.0 mm × 100 mm, 3.5 μm particle size, 80 Å pore size) with a Phenomenex (Torrance, CA, USA) A SecurityGuard C18 guard column (4.0 mm × 3.0 mm) was used for LC-MS/MS analysis. The controlling software for the sample analysis was Analyst 1.7.2 (Applied Biosystems/MDS Analytical Technologies, Foster City, CA, USA). Data analysis was completed using MultiQuantTM 3.0.3 (Applied Biosystems/MDS Analytical Technologies, Foster City, CA, USA).

The HPLC parameters used for the LC-MS/MS analysis of the PITC panel were as follows: solvent A: 0.2% (*v*/*v*) formic acid in water, and solvent B: 0.2% (*v*/*v*) formic acid in acetonitrile. The gradient profile for this UHPLC solvent run was as follows: t = 0 min, 0% B; t = 0.5 min, 0% B; t = 5.5 min, 95% B; t = 6.5 min, 95% B; t = 7.0 min, 0% B; and t = 9.5 min, 0% B. The column oven was set at 50 °C. The flow rate was 500 μL/min, and the sample injection volume was 10 μL. The mass spectrometer was set to a positive electrospray ionization mode with a scheduled multiple reaction monitoring (MRM) scan. The IonSpray voltage was set at 5500 V and the temperature at 500 °C. The curtain gas (CUR), ion source gas 1 (GAS1), ion source gas 2 (GAS2), and collision gas (CAD) were set at 20, 40, 50, and medium, respectively. The entrance potential (EP) was set to 15 V. The declustering potential (DP), collision energy (CE), collision cell exit potential (CXP), MRM precursor ion (Q1), and fragment ion (Q3) were optimized and set individually for each analyte and isotope-labeled ISTD.

For DFI-MS/MS analysis, the UHPLC autosampler was connected directly to the MS ion source via red PEEK tubing. The DFI buffer mentioned above was used as the mobile phase. The flow rate was programmed as follows: t = 0 min, 30 μL/min; t = 1.6 min, 30 μL/min; t = 2.4 min; 200 μL/min; t = 2.8 min, 200 μL/min; and t = 3.0 min, 30 μL/min. The sample injection volume was 20 μL. The IonSpray voltage was set at 5500 V and the temperature was set at 200 °C. The CUR, GAS1, GAS2, and CAD were set at 20, 40, 50, and medium, respectively. The EP and CXP were set at 10 and 15 V, respectively, for positive mode and −10 and −15 V, respectively, for negative mode. Likewise, the DP, CE, Q1, and Q3 were optimized and set individually for each analyte and ISTD.

For the separation of organic acids by LC-MS/MS, the HPLC solvents used were (A) 0.01% (*v*/*v*) formic acid in water and (B) 0.01% (*v*/*v*) formic acid in acetonitrile. The gradient profile was as follows: t = 0 min, 25% B; t = 6.0 min, 65% B; t = 6.3 min, 90% B; t = 6.5 min, 100% B; t = 7.0 min, 100% B; t = 7.5 min, 25% B; t = 12.0 min, 25% B. The column oven was set to 40 °C. The flow rate was 400 μL/min, and the sample injection volume was 10 μL. The mass spectrometer was set to a negative electrospray ionization mode with scheduled MRM scanning. The IonSpray voltage was set at −4500 V and the temperature at 400 °C. The CUR, GAS1, GAS2, and CAD were set at 20, 30, 30, and medium separately. The EP was set at −10 V, and the DP, CE, CXP, Q1, and Q3 were optimized and set individually for all the analytes and isotope-labeled ISTDs.

For data analysis, it was performed with MetaboAnalyst 6.0. Metabolites with more than 20% of missing values were excluded from further analysis. After the filtering step, there were 311 missing values (6.8% of the data, Kruskal-Wallis test: **p = 0.735**). The percentage of missing values or average abundance did not differ significantly across experimental groups, ruling out batch effects.
